# Proton Pump Inhibitor Omeprazole Alters the Spiking Characteristics of Proteinoids

**DOI:** 10.1021/acsomega.4c10790

**Published:** 2025-01-27

**Authors:** Panagiotis Mougkogiannis, Andrew Adamatzky

**Affiliations:** Unconventional Computing Laboratory, University of the West of England, Bristol BS16 1QY, U.K.

## Abstract

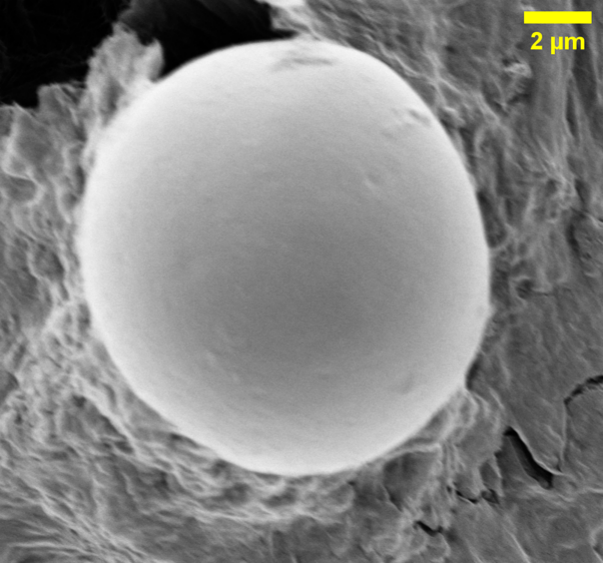

This study reveals the significant effect of the proton pump inhibitor omeprazole on the spiking behavior of proteinoids, leading to a transformative shift in the field of unconventional computing. Through the application of different concentrations of omeprazole, we see a notable modification in the spiking characteristics of proteinoids, including significant alterations in amplitude, frequency, and temporal patterns. By using Boolean logic techniques, we analyze the complex dynamics of the proteinoid-omeprazole system, revealing underlying patterns and connections that question our understanding of biological computing. Our research reveals the unexplored potential of proteinoids as a foundation for unconventional computing. Moreover, our research indicates that the electrical spiking observed in proteinoids may be linked to the movement of protons. This discovery offers new insights into the fundamental mechanisms governing the spiking activity of proteinoids, presenting promising opportunities for future research in this area. Additionally, it opens up possibilities of developing new computational models that exploit the inherent nonlinearity and complexity of biological systems. By combining the effects of omeprazole-induced spikes with Boolean logic, a wide range of opportunities arise for information processing, pattern identification, and problem-solving. This pushes the limits of what can be achieved with bioelectronics.

## Introduction

Neuronal spiking, which is the primary mechanism for processing information in biological neural networks, has been the focus of extensive scientific study for many years.^1^ It has expanded outside the field of neurobiology, serving as a source of inspiration for the development of artificial neural networks (ANNs) that attempt to replicate the computational efficiency and adaptability of biological systems.^2^ As our knowledge of brain dynamics grows, there is an increasing fascination with developing artificial neurons that closely resemble biological ones and can demonstrate spiking behavior.^3^ Proteinoids have been identified as a promising material for building biological neural networks.^4^ The thermal proteins, first developed by Fox and his team in the 1960s, have demonstrated the ability to produce microspheres that can display a range of biologically inspired responses. These behaviors include osmotic swelling, budding, and electrical activity similar to the firing of neurons.^5^ Proteinoids possess the capacity to autonomously generate structures that exhibit characteristics similar to neurons, making them a compelling candidate for bottom-up methodologies in the field of biological intelligence and research into the origins of life.^6^ Recent research has shown that proteinoid microspheres are capable of producing electrical spikes when exposed to certain stimuli, such as changes in pH, temperature, and particular molecules.^7−9^ The spiking activity, although not exactly the same as that of biological neurons, exhibits numerous important features, such as all-or-nothing responses and refractory periods.^10^ The spiking proteinoid networks have a wide range of potential applications, including bioinspired computing and innovative drug screening platforms.^11,12^ As the study of proteinoid-based artificial neural networks advances, it is crucial to understand the potential interactions between these systems and commonly used pharmaceuticals. Drugs that can modify ion transport across membranes are of special interest because they have a capacity to disrupt the mechanisms that cause proteinoid spiking activity.^13^ Omeprazole, a proton pump inhibitor (PPI), is one such drug that warrants investigation in this context. Omeprazole is commonly given to treat acid-related gastrointestinal disorders. It works by blocking the H^+^/K^+^-ATPase enzyme in gastric parietal cells, which effectively reduces the release of gastric acid.^14^ Given the widespread use of omeprazole and other proton pump inhibitors (PPIs) by millions of patients globally, and the fact that they are often taken for long durations, it is important to investigate the possibility of off-target effects on both biological and artificial brain systems.^15^ Although the fundamental function of omeprazole is widely understood in relation to gastric physiology, its impact on other systems, especially those related to ion transport and electrical activity, is still not fully known.^16^ Considering the increasing fascination with proteinoid-based artificial neural networks and their potential uses in biomedical research and drug development, it is essential to examine whether commonly prescribed drugs such as omeprazole could inadvertently affect the functioning of these systems.

**Figure 1:**
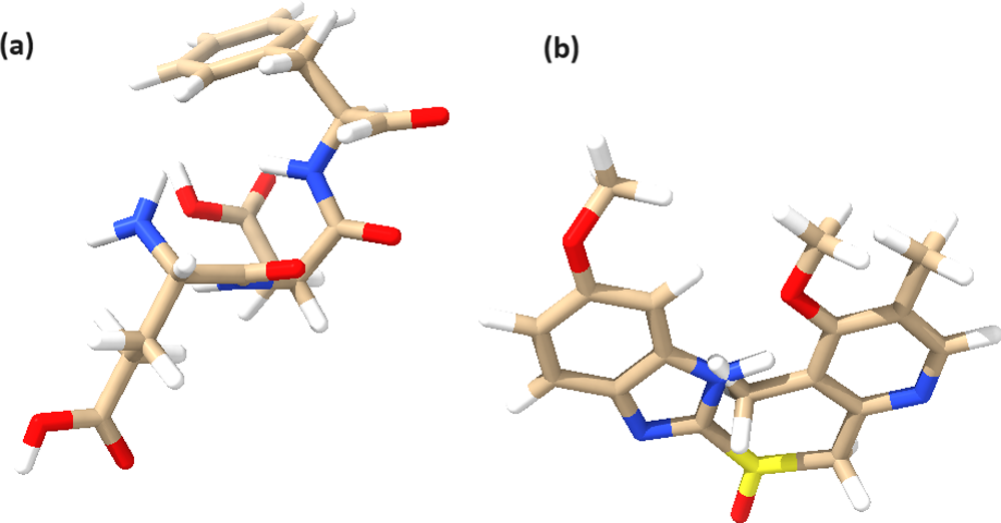
Structural representations of (a) l-Glu:l-Phe:l-Asp proteinoid and (b) omeprazole. The proteinoid structure shown is a simplified motif. It illustrates basic chemical connectivity. The actual samples likely contain more complex and varied arrangements of these amino acids. The AMBER force field optimized the molecular geometry. It is a classical molecular mechanics method. It gives a good first approximation of the structure’s local conformation. The proteinoid structure showcases the amino acid composition and the peptide bonds linking the monomers. The omeprazole structure is depicted with different colors representing different atoms: carbon (gray), hydrogen (white), nitrogen (blue), oxygen (red), and sulfur (yellow). Omeprazole is a chiral compound with a racemic mixture, having a molar mass of 345.42 g mol^–1^ and a melting point of 156 °C (313 °F). The density of omeprazole is 1.4 ± 0.1 g/cm^3^. CHIMERA allows for interactive visualization of the 3D model of omeprazole, enabling a full understanding of its spatial organization and molecular structure.^17^

The structural representations of l-Glu:l-Phe:l-Asp proteinoid and omeprazole are depicted in Figure 1. The proteinoid structure (Figure 1a) highlights the amino acid composition and the peptide bonds linking the monomers, while the omeprazole structure (Figure 1b) is represented with different colors for each atom type.

The Izhikevich model of thalamocortical neurons offers excellent foundation for understanding complex neural dynamics.^18^ Nevertheless, in order to fully understand the possible impacts of pharmaceuticals such as omeprazole on systems resembling neurons, it is essential to expand our perspective beyond conventional neuronal models. Proteinoids, due to their capacity to display spiking activity, provide a fascinating platform for investigating these interactions. The electrical activity of proteinoids may not precisely align with the dynamics outlined in the Izhikevich model. However, by comparing it to established brain models, we can get insights into the potential use of proteinoids as biomimetic computing components. The inclusion of omeprazole into this system introduces an additional level of complexity. The Izhikevich neuron model, particularly its thalamocortical variant, has been widely used to simulate realistic neuronal spiking patterns due to its computational efficiency and biological plausibility.^18^ This model captures the dynamics of thalamocortical neurons, which play a crucial role in sensory processing and cortical oscillations. The thalamocortical Izhikevich neuron is described by a system of two differential equations:
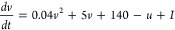
1
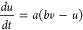
2where *v* represents the membrane potential, *u* is a recovery variable, *I* is the input current, and *a*, *b* are parameters that can be adjusted to reproduce various firing patterns observed in thalamocortical neurons. When *v* reaches a peak of +30 mV, the following reset condition is applied: *v* ← *c*, *u* ← *u* + *d*, where *c* and *d* are additional parameters. This model can reproduce a wide range of spiking behaviors, including regular spiking, intrinsically bursting, and low-threshold spiking, which are characteristic of different types of thalamocortical neurons.^18^ This study will determine if proteinoid networks, when influenced by omeprazole, show changes in their dynamics that are similar to changes in parameters of the Izhikevich model, such as the recovery rate (parameter ‘a’) or the sensitivity of the recovery variable (parameter ‘b’). These findings could not only improve our awareness of proteinoid-based artificial neural networks but also offer novel insights into the wider neurological implications of commonly prescribed drugs like as omeprazole.

This study seeks to fill this gap in knowledge by conducting a systematic investigation into the impact of omeprazole on the spiking characteristics of proteinoid microspheres. Our objective is to investigate the effects of omeprazole, at concentrations commonly used in medical treatment, on proteinoid preparations. We will analyze any resulting changes in electrical activity to determine whether there are any interactions that could affect the functioning of proteinoid-based artificial neural networks.

## Results and Discussion

### Morphological Characterization of Proteinoids Using Scanning Electron Microscopy

**Figure 2:**
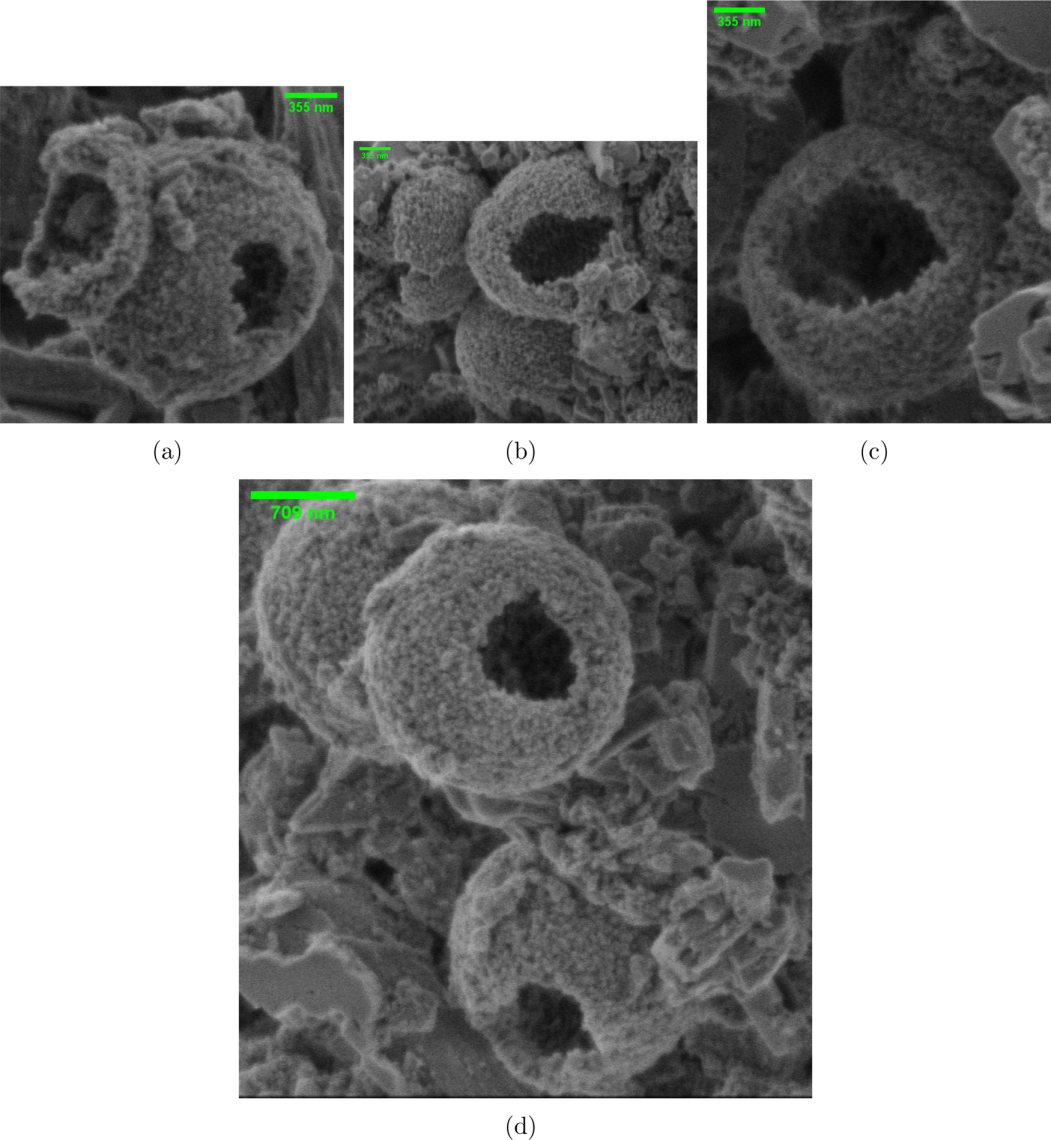
Scanning electron microscopy (SEM) images of proteinoid microspheres showcasing their hollow structures and dimensions. (a) A proteinoid microsphere with a diameter of 1792 nm, featuring a hollow cavity with a diameter of 638 nm. An adjacent open microsphere with a diameter of 1152 nm is also visible. (b) Multiple proteinoid microspheres with varying dimensions: a microsphere with a diameter of 1889 nm and a hollow diameter of 1228 nm, accompanied by two other microspheres measuring 1236 and 1412 nm in diameter. (c) A proteinoid microsphere with a diameter of 1853 nm, exhibiting a hollow structure with a diameter of 1195 nm. (d) A proteinoid microsphere measuring 1793 nm in diameter, containing a hollow cavity with a diameter of 689 nm. The presence of these hollow structures within the proteinoid microspheres highlights their potential for encapsulation and controlled release applications in various fields, such as drug delivery and biomedical research.

The scanning electron microscopy (SEM) images in Figure 2 depict the complex form and hollow structures of the proteinoid microspheres that were produced using l-glutamic acid, l-aspartic acid, and l-phenylalanine. The hollow microspheres are formed through a self-assembly process that is influenced by the distinctive characteristics of the amino acids and the presence of potassium nitrate (KNO_3_) as an inert electrolyte during synthesis. The proteinoid microspheres display a variety of sizes, with diameters ranging from 1152 to 1889 nm, as depicted in Figure 2a–d. The microspheres have hollow cavities of diverse sizes, ranging from 638 to 1228 nm. In Figure 2a, the presence of a neighboring open microsphere allows for a distinct observation of the empty internal arrangement. The growth of empty structures within the proteinoid microspheres can be seen as a type of morphogenesis, in which the spontaneous arrangement of the amino acid building blocks results in the emergence of specific structural characteristics. The hydrophobic interactions between the amino acid side chains, especially those of l-phenylalanine with its hydrophobic benzyl group, are likely to have an impact on this process. Hydrophobic interactions facilitate the organization of amino acids into spherical structures, where the hydrophilic parts are positioned on the outside and the hydrophobic parts remain hidden within the interior. Moreover, the addition of KNO_3_ as an inert electrolyte during the synthesis process could potentially facilitate the generation of hollow structures. The electrolyte has the ability to impact the way charges are distributed and the strength of ions in the solution, which in turn affects how amino acids assemble together and arrange themselves. The use of a KNO_3_ concentration of 0.065 mol/L in this study is expected to offer an ideal equilibrium between charge screening and ionic interactions, hence promoting the formation of stable hollow microspheres. The inherent hollowness of the proteinoid microspheres emphasizes their potential for use in encapsulation and controlled release applications. The hollow spaces inside the microspheres can be used as storage areas for different substances, such as medications, enzymes, or other biologically active molecules. The varying dimensions of the hollow cavities, ranging from 638 to 1228 nm, indicate their ability to control a wide range of material sizes. This adaptability makes them suitable carriers for a variety of applications.^19−21^

### Investigating Omeprazole’s Influence on Proteinoid Spiking Behavior

**Figure 3:**
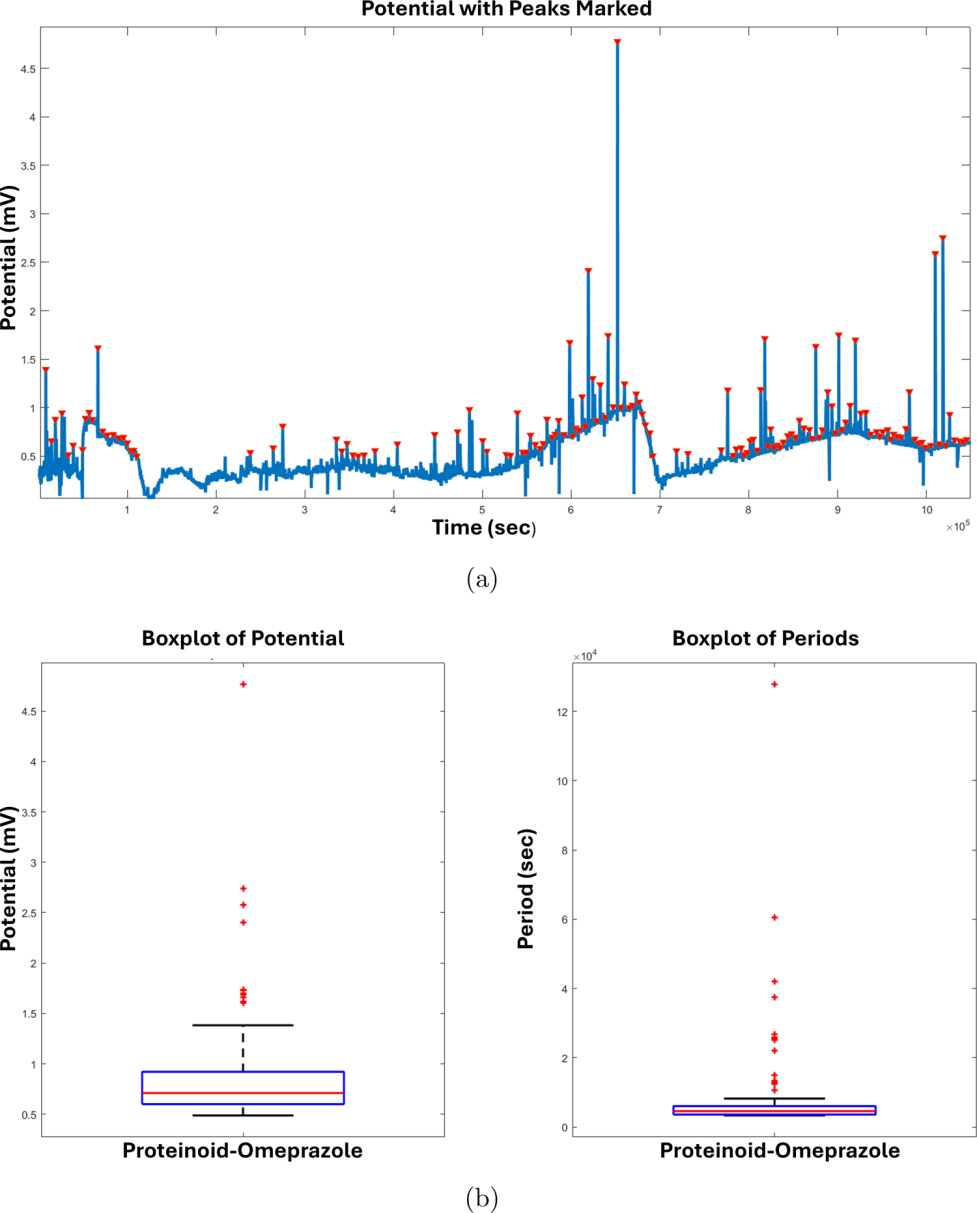
Electrical potential measurements of the proteinoid-omeprazole system. (a) The graph displays the electrical potential (mV) recorded over time (s), with distinct spikes marked by red triangles. These spikes indicate significant fluctuations in the electrical potential, suggesting the occurrence of dynamic events within the system. (b) Box plots representing the statistical distribution of the spike amplitudes (left) and spike periods (right). The amplitude box plot reveals a median value of 0.71 mV, with quartiles at 0.60 and 0.92 mV. The mean amplitude is 0.85 mV, and the standard deviation is 0.51 mV. Notably, there is an outlier at 4.77 mV, indicating an exceptionally high-amplitude event. The period box plot shows a median value of 4591.00 s, with quartiles at 3609.25 and 6038.75 s. The mean period is 7520.47 s, and the standard deviation is 12,710.38 s. The presence of an extreme outlier at 127,866.00 s suggests the occurrence of an extraordinarily long interspike interval.

The electrical potential measurements of the proteinoid-omeprazole system, as shown in Figure 3, provide valuable insights into the dynamic behavior and underlying mechanisms of this complex system. In this section, we investigated the effects of the highest dosage of omeprazole at 1.30 mg/mL on the electrical properties of proteinoids. The time-varying potential recordings in Figure 3a display clear spikes, denoted by red triangles, that signify notable variations in the electrical potential. The presence of these spikes indicates the occurrence of dynamic events within the system, such as the formation or dissociation of proteinoid-omeprazole complexes, changes in structure, or processes involving the transfer of electric charge. The statistical examination of the spike amplitudes and periods, as shown in the box plots in Figure 3b, offers an in-depth overview of the distribution and variability of these electrical events. The box plot of amplitudes shows a median value of 0.71 mV and a mean value of 0.85 mV, showing that most of the spikes have relatively low magnitudes. Nevertheless, the existence of an outlier at 4.77 mV is very remarkable, as it signifies an extremely large-scale event. The significant deviation from the average can be attributed to the discharge of electrical energy caused by the interaction between proteinoid and omeprazole. Capacitive discharge is the process by which an electrical system gradually stores electrical charge, resulting in the accumulation of potential energy. Once the charge reaches up to a critical level, it has the ability to discharge suddenly, causing a sharp rise in electrical potential. The potential value at 4.77 mV is most likely an outlier that indicates a capacitive discharge event, where a large quantity of stored charge is quickly released, resulting in a spike with a high amplitude. The capacitive discharge effects found in the proteinoid-omeprazole system can be affected by various factors, such as the structural characteristics of the proteinoid microspheres, the existence of charged functional groups, and the interactions between the proteinoid and omeprazole molecules. The proteinoid microspheres’ hollow shapes, as observed in the scanning electron microscope images, likely enhance the capacitive behavior by forming a restricted area for the accumulation and storage of charges. In addition, the box plot in Figure 3b illustrates the range of values for the interspike intervals, with a median of 4591.00 s and a mean of 7520.47 s. The existence of an exceptional outlier at 127866.00 s indicates the presence of an exceptionally lengthy interspike gap. This anomaly may suggest an extended time of charging, during which the system steadily gathers charge until it reaches the threshold for capacitive discharging. The presence of capacitive discharge effects and the accompanying high-amplitude spikes in the proteinoid-omeprazole system (Table 1) have important consequences for prospective uses. These electrical occurrences can be used for different objectives, such as triggering the release of encapsulated drugs or regulating the function of bioactive compounds. The capacity to regulate and control the discharge behavior of the proteinoid-omeprazole system presents opportunities for the advancement of stimuli-responsive drug delivery systems or biosensors that respond to inputs.

**Table 1:** Table Provides a Statistical Study of Proteinoid-Omeprazole Electrical Potential Spikes^a^

parameter	amplitude (mV)	period (s)
quartiles	0.60, 0.71, 0.92	3609.25, 4591.00, 6038.75
mean	0.85	7520.47
maximum	4.77	127866.00
minimum	0.49	3339.00
standard deviation	0.51	12710.38

a Quartiles, mean, maximum, minimum, and standard deviation characterize spike amplitude and period. Most spikes had modest amplitudes, with a mean of 0.85 mV and quartiles of 0.60–0.92 mV. The system’s capacitive discharge effects may explain the 4.77 mV maximum amplitude, which suggests a high-amplitude event. The mean interspike interval is 7520.47 s, while the quartiles range from 3609.25 to 6038.75 s. The highest interspike interval of 127,866.00 s suggests a considerable charging duration before a capacitive discharge event. Standard deviations of 0.51 mV for amplitude and 12,710.38 s for period show the proteinoid-omeprazole system’s electrical variability.

**Figure 4:**
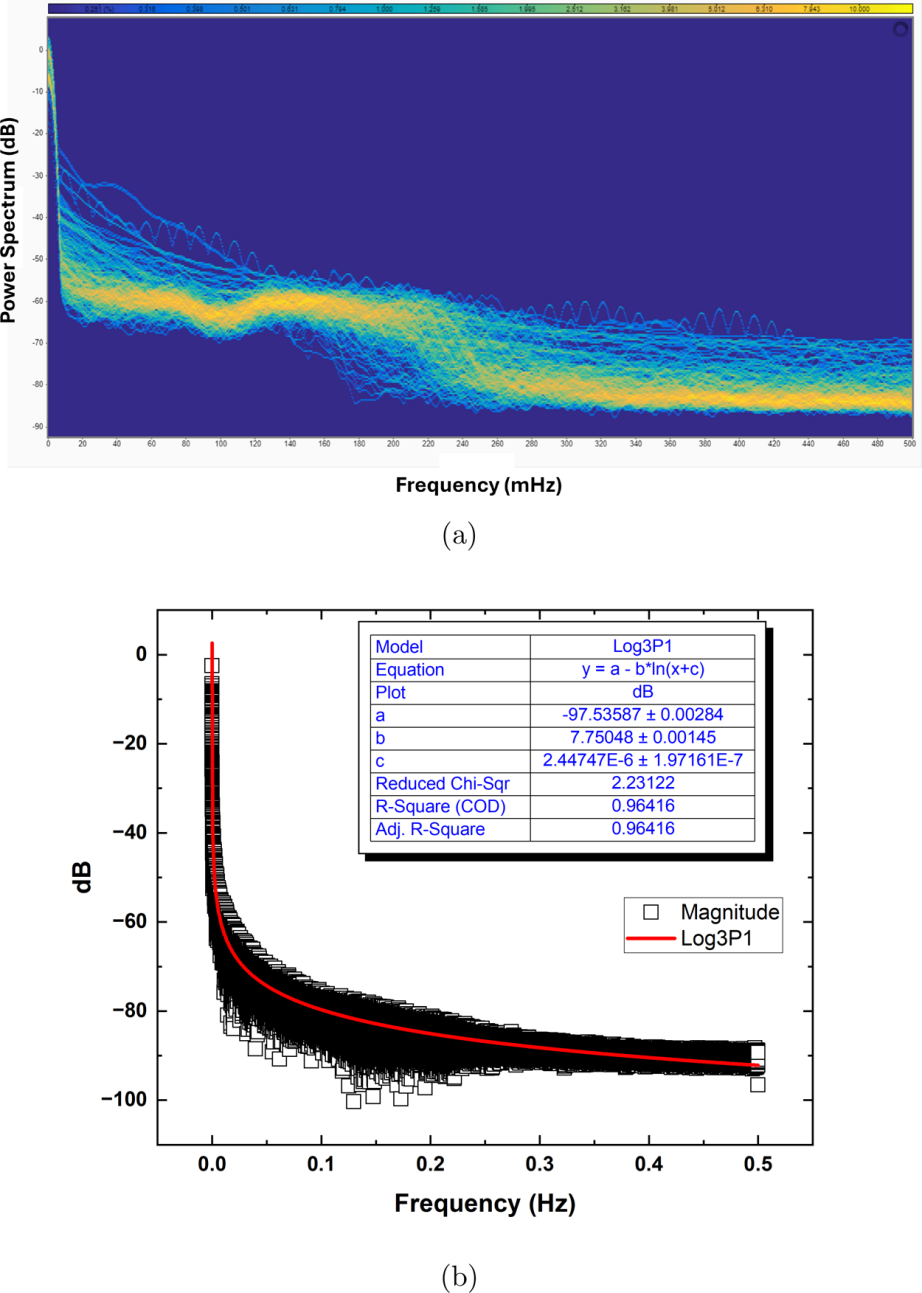
Persistent spectrum and logarithmic fitting of the proteinoid-omeprazole system’s electrical activity. (a) Proteinoid-omeprazole system persistent spectrum, showing magnitude (decibels) and frequency (Hertz). Power law behavior shows that spectrum magnitude decreases with frequency. The magnitude has a mean of −84.22 dB, a median of −87.34 dB, and a standard deviation of 7.94 dB. Q1, Q2, and Q3 are −89.53, −87.34, and −81.56 dB. The equation for logarithmic fitting of the persistent spectrum using the Log3P1 model: *y* = *a* – *b* ln(*x* + *c*). In this equation, *y* represents the magnitude in decibels (dB), *x* denotes frequency in Hertz (Hz), and *a*, *b*, and *c* are fitting parameters. The fitting parameters are *a* = −97.53587 ± 0.00284, *b* = 7.75048 ± 0.00145, and *c* = (2.44747 ± 1.97161) × 10^–6^ A decreased chi-square value of 2.23122 and an *R*-square (COD) value of 0.96416 suggest significant agreement between experimental data and the logarithmic model. The high adjusted *R*-square value of 0.96416 further confirms the goodness of fit. The persistent spectrum’s power law behavior shows that the proteinoid-omeprazole system exhibits pink noise and operates near a critical state between order and chaos.

The findings shown in Figure 4 offer significant understanding into the electrical behavior and critical dynamics of the proteinoid-omeprazole system. The persistent spectrum, depicted in Figure 4a, demonstrates a distinct power law pattern, with the magnitude diminishing as the frequency rises. This characteristic is an indicator of pink noise and suggests the system is functioning in close proximity to a critical state that lies between order and chaos.^22,23^ The logarithmic fitting of the persistent spectrum, as shown in Figure 4b, demonstrates a robust agreement between the empirical data and the Log3P1 model. The parameter *b*, with an exact value of 7.75048 ± 0.00145, plays a role in detecting how to differentiate between order and chaos within the system. The literature states that the value of *b* is connected to the power law exponent β by the equation β = −*b*.^24,25^ The Log3P1 model’s logarithmic fit of the persistent spectrum reveals key traits of our system’s frequency response. The high *R*-square value (>0.96) shows the logarithmic model fits well. It captures the link between frequency and magnitude. This suggests the proteinoid network’s response follows a natural logarithmic decay. The small uncertainty in the primary fitting parameters (<1%) demonstrates the robustness of this relationship. The near-zero value of parameter *c* is important. It is (2.44747 ± 1.97161) × 10^–6^. It means the system’s response begins to decay immediately from the initial frequency, without an offset. These traits support our hypothesis about the network’s frequency-dependent behavior and its impact on signal processing.

Within the framework of critical dynamics, the power law exponent β provides understanding of the fundamental mechanisms and features of the system’s behavior. Pink noise, with a power law exponent near −1, indicates that the system is at a critical condition, maintaining an unstable equilibrium between order and chaos.^26^ The value of *b* obtained in this situation indicates that the proteinoid-omeprazole system has pink noise characteristics and operates in close proximity to criticality. The existence of pink noise and critical dynamics in the proteinoid-omeprazole system has significant implications. Systems functioning in close proximity to criticality are recognized to display self-organized criticality, when the system naturally progresses toward a critical state without requiring external adaptations.^27^ Self-organized criticality is linked to the development of complex patterns of behavior, long-range correlations, and the capacity to process information effectively.^28,29^ Moreover, the presence of power law behavior and the specific value of *b* indicate that the proteinoid-omeprazole system might have the ability to perform complex computations. Systems operating in close proximity to criticality have been demonstrated to display optimal information processing, heightened sensitivity to external stimuli, and the capacity to generate a wide range of responses.^30,31^ These features are essential for the advancement of innovative bioelectronic devices and for understanding of biological information processing.

#### Thalamocortical Stimulation of Proteinoids and Omeprazole-Proteinoid Mixture Using Izhikevich Neuron Model

This subsection examines the reaction of proteinoids and a mixture of omeprazole-proteinoid to thalamocortical stimulation using the Izhikevich neuron model. The Izhikevich neuron model is a highly efficient and biologically realistic model that accurately represents the spiking behavior of thalamocortical neurons.^18^ In order to examine the effects of thalamocortical stimulation, we created input signals that imitate the firing patterns of thalamocortical neurons using the Izhikevich model. The input signals were subsequently fed to samples of both pure proteinoid and omeprazole-proteinoid mixture, and their corresponding responses were recorded. The input channel, which displays the output of the Izhikevich neuron model, is plotted together with the corresponding output channels, enabling a direct comparison of the patterns of stimulation and response.

**Figure 5:**
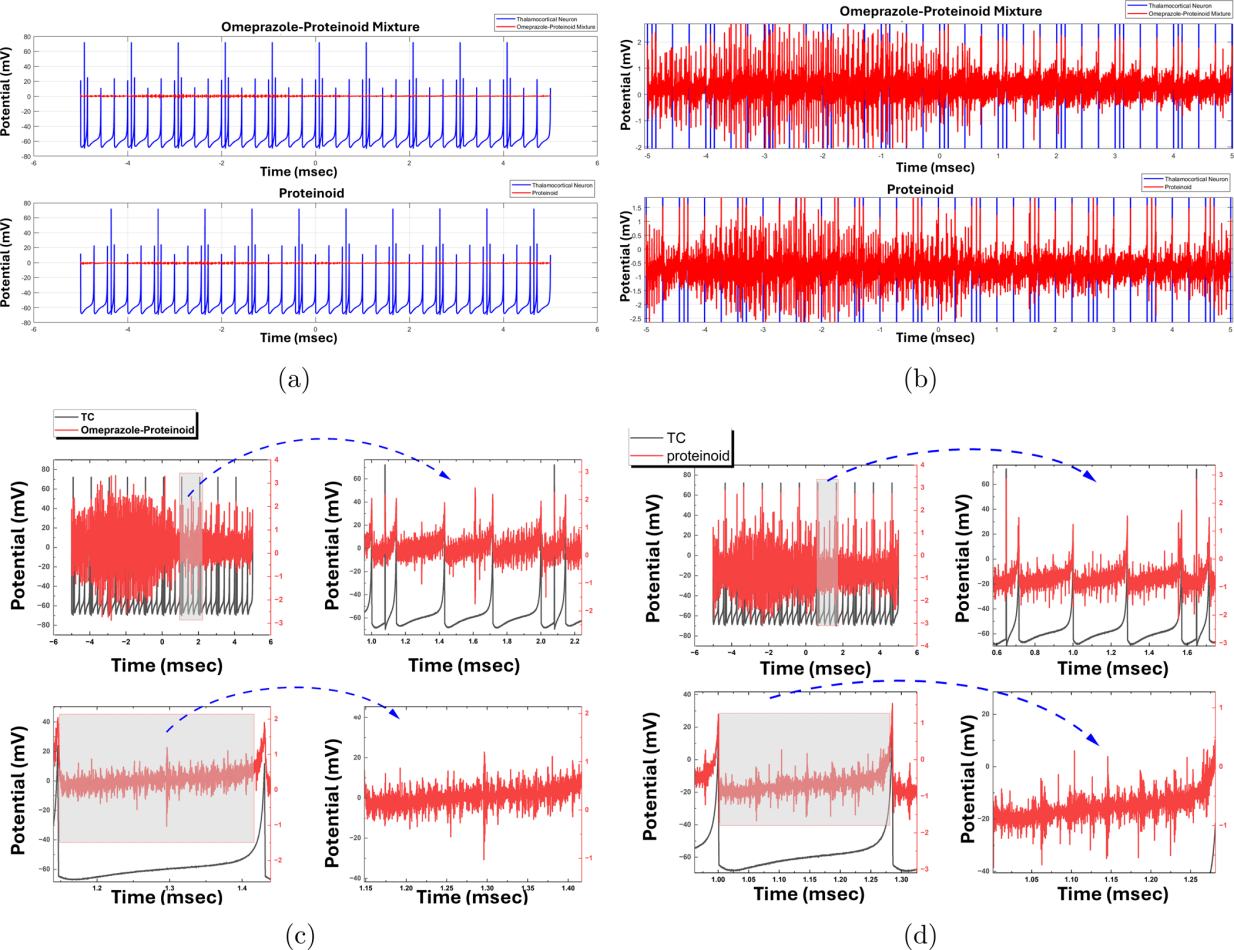
Figure shows the impact of thalamocortical stimulation on both an omeprazole-proteinoid mixture and pure proteinoid, and examines the resulting responses. (a) The graph displayed in blue shows the thalamocortical stimulation produced using the Izhikevich neuron model, while the red graph illustrates the response of the omeprazole-proteinoid mixture (b) The blue graph depicts the thalamocortical stimulation generated using the Izhikevich neuron model, while the red graph represents the response of the -proteinoid sample. The thalamocortical stimulation displays a fluctuating pattern of spiking activity, characterized by changes in both amplitude and frequency. The combined effect of omeprazole and proteinoid reacts to the stimulation of the thalamocortical systems with an average electrical potential of 0.31 mV and a measure of variability of 0.38 mV. The response exhibits both positive and negative deviations, reaching a peak value of 3.33 mV and a lowest value of −2.87 mV. In contrast, the pure proteinoid response to the same thalamocortical stimulation has a lower mean potential of −0.65 mV and a similar standard deviation of 0.38 mV. The proteinoid response exhibits both positive and negative deviations, ranging from a maximum value of 3.02 mV to a minimal value of −3.06 mV. (c) Enlargement of the proteinoid-omeprazole spiking response, showing the “remembering” region induced by the TC stimulation. The TC stimulation is represented in gray, while the proteinoid spiking is shown in red. (d) Enlargement of the proteinoid spiking response, indicating the TC-induced “remembering” area. TC stimulation is gray, proteinoid spiking is red.

In Figure 5, the impact of thalamocortical (TC) stimulation on an omeprazole-proteinoid mixture and pure proteinoid is investigated, providing valuable insights into their respective responses. The TC stimulus, produced via the Izhikevich neuron model, displays a dynamic pattern of spiking activity characterized by fluctuations in both amplitude and frequency. The combination of omeprazole and proteinoid exhibits a distinct response to TC stimulation, characterized by an average potential of 0.31 mV and a standard variation of 0.38 mV. The response demonstrates significant variations, with a peak value of 3.33 mV and a plateau value of −2.87 mV. This implies that the presence of omeprazole in the proteinoid system could lead to a wider variety of electrical responses, which may indicate a regulatory influence on the system’s ability to be stimulated and its overall behavior. Conversely, the pure proteinoid demonstrates a decreased average potential of −0.65 mV when subjected to the same TC stimulation, while maintaining a similar standard deviation of 0.38 mV. The proteinoid response exhibits both positive and negative deviations, ranging from a maximum of 3.02 mV to a minimum of −3.06 mV. Although the broad range of responses is comparable to that of the omeprazole-proteinoid mixture, the negative mean potential indicates that the pure proteinoid may have a somewhat different baseline electrical activity or a separate response profile to the TC stimulation. Figure 5c,d display magnified perspectives of the spiking reactions observed in the omeprazole-proteinoid mixture and pure proteinoid, respectively. The larger parts emphasize the existence of a “remembering” region that is caused by the TC stimulation. The TC stimulation, depicted in gray, produces a distinct pattern of proteinoid spiking activity, illustrated in red. The region associated with memory indicates that the proteinoid systems, whether with or without omeprazole, display a type of short-term memory or sustained activity in response to TC activation. This event suggests that proteinoids have the capacity to store and preserve information from external stimuli, which could serve as a basis for future studies on memory formation and information processing in these systems.

**Figure 6:**
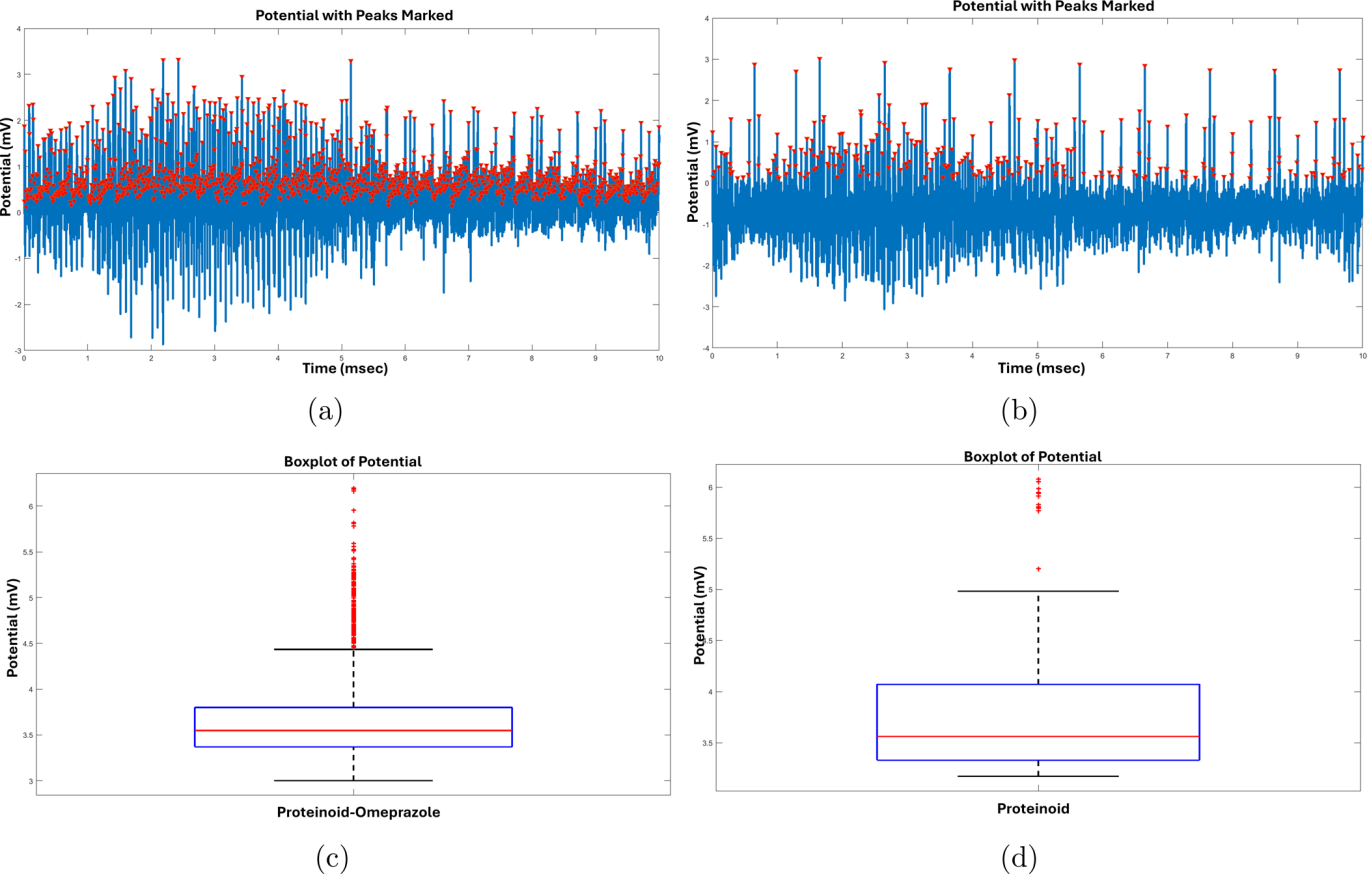
(a) Potential (mV) vs time (ms) plot for the omeprazole-proteinoid system, with inverted triangles indicating the detected spikes. The spikes were identified using a peak detection program with a threshold of 0.1 mV and a minimum peak distance of 50 ms. The plot reveals the occurrence of distinct electrical spikes in the omeprazole-proteinoid system. (b) Potential (mV) vs time (ms) plot for the proteinoid system, with inverted triangles indicating the detected spikes. The spikes were identified using the same peak detection program and parameters as in (a). The plot demonstrates the presence of electrical spikes in the proteinoid system. (c) Amplitude statistics for the omeprazole-proteinoid system under thalamocortical stimulation. The quartiles of the spike amplitudes are 3.37, 3.55, and 3.80 mV, with a mean amplitude of 3.69 mV. The maximum amplitude observed is 6.20 mV, while the minimum amplitude is 3.00 mV. The standard deviation of the amplitudes is 0.51 mV, indicating the variability in the spike magnitudes. (d) Amplitude statistics for the proteinoid system under thalamocortical stimulation. The quartiles of the spike amplitudes are 3.33, 3.56, and 4.07 mV, with a mean amplitude of 3.80 mV. The maximum amplitude observed is 6.08 mV, while the minimum amplitude is 3.17 mV. The standard deviation of the amplitudes is 0.64 mV, suggesting a slightly higher variability compared to the omeprazole-proteinoid system.

Figure 6 displays an extensive look of the electrical potential measurements and statistical comparisons between the omeprazole-proteinoid and proteinoid systems during thalamocortical stimulation. The potential vs time charts in Figure 6a,b demonstrate the presence of separate electrical spikes in both systems, with inverted triangles denoting the observed spikes. The spikes were detected using a peak detection algorithm that had a threshold of 0.1 mV and a minimum peak distance of 50 ms. This ensured that only important electrical events were captured while reducing the impact of noise. Upon careful analysis of the amplitude statistics in Figure 6c,d, significant differences between the omeprazole-proteinoid and proteinoid systems became apparent. The omeprazole-proteinoid system has an average spike amplitude of 3.69 mV, with quartiles of 3.37, 3.55, and 3.80 mV. On the other hand, the proteinoid system has a marginally greater average amplitude of 3.80 mV, with quartiles at 3.33, 3.56, and 4.07 mV. These findings indicate that the presence of omeprazole in the proteinoid system may alter the electrical spiking behavior, perhaps affecting the intensity of the spikes. Remarkably, the omeprazole-proteinoid system exhibits a more limited range of spike amplitudes in comparison to the proteinoid system alone. The omeprazole-proteinoid system has a smaller standard deviation of 0.51 mV, compared to the proteinoid system’s standard deviation of 0.64 mV. This result provides support for the claim. The narrower distribution suggests that the omeprazole-proteinoid system demonstrates more stable and less fluctuating spike amplitudes, which may indicate that omeprazole has a stabilizing influence on the electrical activity. Examining the spike amplitudes’ extreme values can provide more insights. The omeprazole-proteinoid system exhibits a peak amplitude of 6.20 mV, slightly above the proteinoid system’s maximum of 6.08 mV. This implies that the presence of omeprazole might promote the production of spikes with somewhat greater amplitude in specific cases. However, the lowest amplitudes of the omeprazole-proteinoid system and the proteinoid system are identical, with values of 3.00 and 3.17 mV respectively. This suggests that both systems have a similar bottom limit for the magnitudes of spikes. The quartile analysis offers a more thorough examination of the distribution of spike amplitudes. In the omeprazole-proteinoid system, 25% of the spikes have amplitudes that are less than 3.37 millivolts (first quartile), 50% have amplitudes that are less than 3.55 millivolts (median), and 75% have amplitudes that are less than 3.80 millivolts (third quartile). In comparison, the proteinoid system demonstrates somewhat higher values for the respective quartiles: 3.33, 3.56, and 4.07 mV. These findings indicate that the proteinoid system exhibits a wider spectrum of spike amplitudes, with a greater percentage of spikes occurring in the higher amplitude range compared to the omeprazole-proteinoid system. The differences in the electrical spiking patterns between the omeprazole-proteinoid and proteinoid systems when subjected to thalamocortical stimulation give rise to interesting questions regarding the fundamental mechanisms at play. Omeprazole, a proton pump inhibitor, can alter the movement of ions and the dynamics of membrane potential in the proteinoid system, resulting in the observed variations in spike amplitudes and distribution. Additional research on the precise interactions between omeprazole and the proteinoid components, as well as their possible effects on ion channels and signaling pathways, could offer useful understanding of the molecular mechanisms behind these electrical events. The results depicted in Figure 6 demonstrate the potential of proteinoid systems as innovative biomaterials for interacting with neurological systems, as well as the influence of omeprazole in modifying their electrical characteristics. The comparative analysis of the omeprazole-proteinoid and proteinoid systems provides a basis for further investigation into the physiological importance of these electrical spikes and their relevance to neuroscience research and bioelectronic applications. Researchers can explore novel possibilities in building intelligent biomaterials and inventing unique methods to modify and interact with brain activity by understanding the elements that influence spiking behavior and the effects of pharmacological drugs such as omeprazole.

#### Quantitative Analysis of Omeprazole Dosage Effects on Proteinoid Spike Characteristics

**Figure 7:**
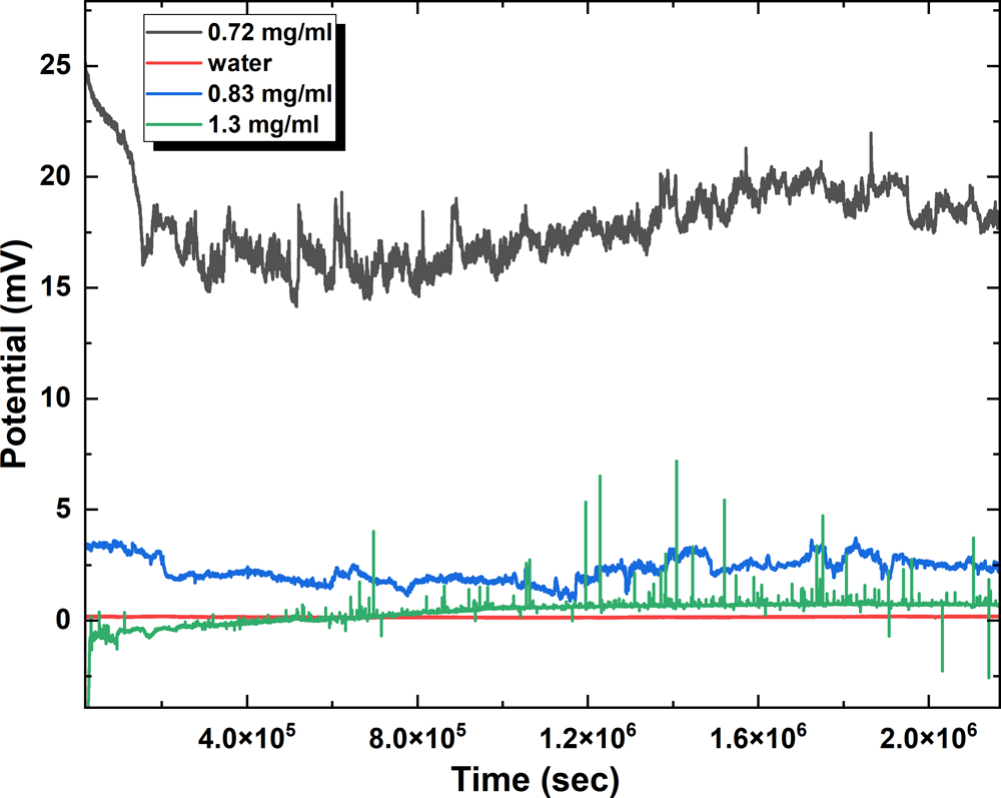
Raw potential values of proteinoids mixed with different concentrations of omeprazole and the blank. The figure presents the time-dependent potential measurements (mV) for proteinoids in the presence of various concentrations of omeprazole (0.72, 0.83, and 1.30 mg/mL) and the blank (no sample). The potential values are plotted against time (s) for a total duration of 24,8 days. The raw data reveals distinct patterns and characteristics for each condition. The proteinoid blank exhibits a relatively stable potential baseline with minimal fluctuations. In contrast, the addition of omeprazole to the proteinoids results in the emergence of potential spikes of varying amplitudes and frequencies. The 0.72 mg/mL omeprazole-proteinoid mixture shows prominent potential spikes with higher amplitudes compared to the other concentrations. As the omeprazole concentration increases to 0.83 and 1.30 mg/mL, the potential spikes become less pronounced and more frequent. These observations suggest a concentration-dependent interaction between omeprazole and proteinoids, leading to alterations in the electrochemical behavior of the system. The raw potential data provides a foundation for further analysis and quantification of the amplitude and period of the potential spikes, enabling a deeper understanding of the omeprazole-proteinoid interaction dynamics.

**Figure 8:**
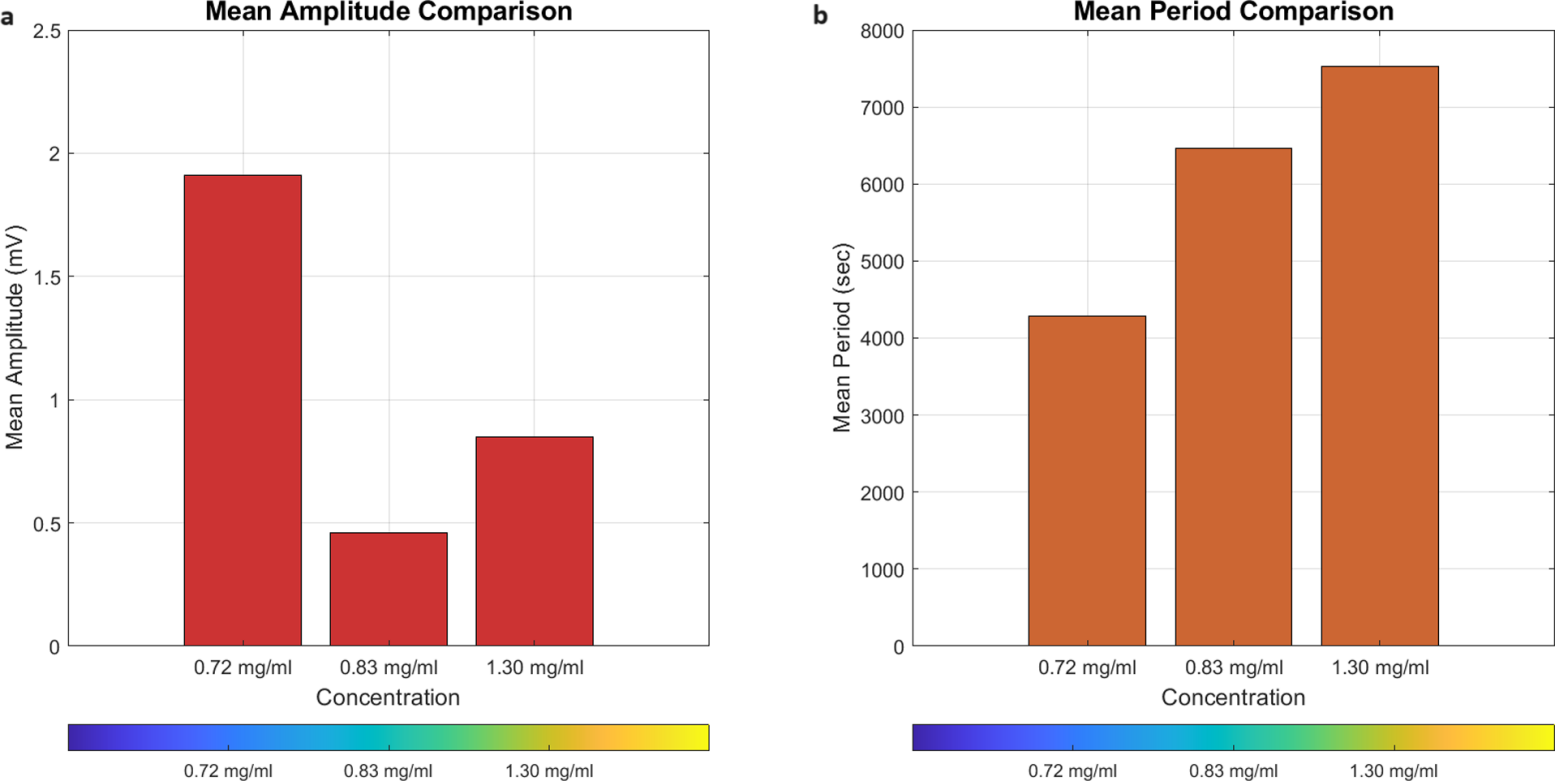
Comparison of mean amplitude and mean period of potential spikes for different dosages of omeprazole-proteinoid. (a) Bar plot depicting the mean amplitude (mV) of potential spikes for omeprazole-proteinoid concentrations of 0.72, 0.83, and 1.30 mg/mL. The mean amplitudes are 1.91, 0.46, and 0.85 mV, respectively. (b) Bar plot representing the mean period (sec) of potential spikes for the same omeprazole-proteinoid concentrations. The mean periods are 4285.28, 6461.62, and 7520.47 s, respectively. The colormap used in both plots indicates the concentration levels, with red corresponding to 0.72 mg/mL, green to 0.83 mg/mL, and blue to 1.30 mg/mL. The plots reveal a decrease in mean amplitude and an increase in mean period as the concentration of omeprazole increases. These findings suggest a concentration-dependent effect on the electrochemical behavior of the omeprazole-proteinoid system, with higher concentrations resulting in lower amplitude and longer period of potential spikes. The results provide insights into the dynamics and stability of the omeprazole-proteinoid system at different dosages, which can have implications for its potential applications in drug delivery and other biomedical fields.

The two figures, Figures 7 and 8, offer significant insights into the impact of omeprazole, a proton pump inhibitor, on the potential spikes produced by proteinoids. The raw information in Figure 7 displays clear patterns and features for each condition. The blank sample shows a relatively consistent baseline potential, whereas the introduction of omeprazole leads to irregular potential (mV) spikes of different sizes and frequencies. Figure 7 presents the potential (mV) versus time (s) plots for omeprazole-proteinoid at concentrations of 0.72 and 0.83 mg/mL. The potential spikes, automatically detected using a MATLAB program, are marked with red inverted triangles. The figure includes statistical data on the amplitude and period of the potential spikes for each concentration, providing insights into the electrochemical behavior and stability of the omeprazole-proteinoid system. Figure 7 displays the potential (mV) versus time (s) and periods (s) for omeprazole-proteinoid at concentrations of 0.72 and 0.83 mg/mL. The potential plots show the variation of potential over time, with potential spikes marked by red inverted triangles.

The statistical analysis in Figure 8 provides additional evidence of the impact of different concentrations of omeprazole on the average intensity and duration of the potential spikes. Omeprazole suppresses potential spikes due to its mechanism of action as a proton pump inhibitor. Omeprazole selectively targets and inhibits the H^+^/K^+^-ATPase enzyme, commonly referred to as the proton pump, which is accountable for the release of hydrogen ions (protons) into the gastric lumen. Omeprazole decreases the acidity of the stomach and raises the pH by blocking the activity of this enzyme. Within the proteinoid system, the potential spikes most likely arise from the interconnected processes of proton and electron transfer that take place at the boundary between the proteinoids and the electrode. The transfer of protons is assisted by the proteinoids’ ability to conduct protons, which can be affected by the presence of proton pump inhibitors such as omeprazole. Upon introduction of omeprazole into the system, it has the potential to interact with the proteinoids and modify their ability to conduct protons. Omeprazole’s ability to hinder proton transfer can result in a decrease in the magnitude of the potential spikes, as depicted in Figure 8. The reduction in amplitude indicates a decrease in the number of protons being transferred across the proteinoid-electrode contact, leading to a diminished magnitude of the potential spikes. Moreover, the rise in the average duration of the potential spikes as the omeprazole concentration increases suggests a decrease in the frequency of the spikes. This effect may be attributed to the reduced availability of protons for transfer due to the inhibition of the proton pump by omeprazole. The increased intervals between spikes indicate a reduced rate of proton transfer and a prolonged duration for the system to recover and produce the next spike. The impact of omeprazole on potential spikes is influenced by its concentration, with higher concentrations leading to greater blockage of the proton pump. Increased concentrations of omeprazole cause a more pronounced inhibition of proton transfer, leading to reduced magnitudes and extended durations of the potential spikes. These findings are significant for comprehending the interaction between proton pump inhibitors and proteinoids, as well as the potential applications of this system in medication delivery and other medicinal domains. The utilization of proton pump inhibitors such as omeprazole to regulate the electrochemical properties of proteinoids presents novel opportunities for precise medication release and targeted delivery mechanisms. Overall, omeprazole suppresses potential spikes in the proteinoid system by inhibiting proton transfer. This results in decreased spike amplitudes and longer spike durations. The effects of concentration on the omeprazole-proteinoid interaction offer useful insights into its dynamics and stability, with possible implications for biological applications.

The choice of concentrations around 0.8 mg/mL was based on omeprazole’s properties and solubility. This concentration balances omeprazole’s solubility limit (about 0.83 mg/mL at pH 7) and its usual therapeutic range. The chosen concentrations (0.72, 0.83, and 1.3 mg/mL) let us observe proteinoid behavior just below, at, and above the solubility limit (0.72 mg/mL). At 1.3 mg/mL, the drug may begin to precipitate. This range shows how the proteinoid-omeprazole interaction changes across these key thresholds. A drug’s solubility in water is key.^32^ It affects its dissolution rate and absorption in the gut. Poorly water-soluble drugs, like omeprazole, are hard to dose. Their low solubility often causes poor, variable bioavailability.^33^ Extensive research has been conducted to explore the factors influencing the aqueous solubility of omeprazole. One key parameter is the gastric volume, which can affect the solubility of the drug. The biopharmaceutics classification system defines highly soluble drugs in adults as those where the maximum dose is soluble in 250 mL of aqueous liquid, equal to the initial gastric volume.^34^ The Dose number (*D*_0_) is the drug solubility, normalized to the dose and gastric volume. It is key to understanding the solubility issues with omeprazole. Interestingly, the gastric volume in children and adults is similar when normalized per kilogram of body weight. However, the drug dosage may differ. This suggests that omeprazole’s solubility may vary with age. It could affect its bioavailability and therapeutic response in different patient groups. Also, controlled gastroretentive delivery systems show promise.^35^ They may solve the solubility limits of omeprazole. These systems aim to extend the time the drug stays in the stomach. This improves solubility and bioavailability, especially for drugs like omeprazole that have pH-dependent solubility.

While the voltage differences appear small, we can confirm their statistical relevance through several measures. The setup uses a PicoLog ADC-24 data logger in differential mode. It provides high-precision measurements with a resolution of 12 μV and an accuracy of ±0.2% for the voltage range used (±2.5 V). This ensures that the observed differences of 0.1–0.2 mV are well within the instrument’s reliable detection capabilities. To confirm that the small voltage changes (Δ*V*) are real biological responses, not measurement artifacts, we include control measurements of pure water oscillations in Figure 7. The control tests show lower amplitude fluctuations (<0.05 mV). This proves the proteinoid-omeprazole signals are distinct from the background noise (σ_noise_).

In order to provide an overview of our findings, we conducted a comparative analysis of omeprazole’s inhibitory capabilities in proteinoids and its impact on other biological systems. This information is summarized in Table 2. Omeprazole slows gastric acid secretion, elevates pH, and lowers proton transfer in proton pumps (H^+^/K^+^-ATPase).^14,36^ This form of inhibition has similarities with the suppression of potential spikes in proteinoids, as both involve a restriction of proton-related activities. Nevertheless, the distinct implications and physiological effects vary between these two systems. Omeprazole exerts inhibitory effects on cytochrome P450 enzymes, resulting in modified drug metabolism, possible drug–drug interactions, and alterations in pharmacokinetics.^37,38^ Although this inhibition is important in the context of drug metabolism, it is distinct from the inhibition characteristics reported in proteinoids, which mainly involve the regulation of proton-related activities and the suppression of potential spikes. Omeprazole demonstrates inhibitory effects in bacterial proton-gated ion channels, which leads to a reduction in bacterial pathogenicity and a potential display of antibacterial capabilities.^39,40^ This inhibition has certain parallels to the suppression of potential spikes in proteinoids, as both entail the regulation of proton-related mechanisms. Nevertheless, the precise consequences and implications of omeprazole inhibition in these two systems are different. Omeprazole furthermore regulates acid-sensing ion channels (ASICs), which are involved in neuroprotection and the control of neuronal excitability.^41,42^ Although both proteinoids and ASICs are influenced by omeprazole in terms of ion channel regulation, their specific actions and physiological consequences vary. The examination of omeprazole’s inhibitory characteristics in various biological systems emphasizes the distinctiveness of its interaction with proteinoids. The findings of our study illustrate that omeprazole has a concentration-dependent effect on proteinoids, leading to the inhibition of potential spikes, decrease in amplitude, and elongation of the spike period. These findings enhance our understanding of the complex relationship between omeprazole and proteinoids and present new opportunities for investigating the potential uses of this system in other domains, such as drug delivery and biological research. Finally, our study on the inhibitory capabilities of omeprazole in proteinoids, together with its comparison to its effects in other biological systems, offers vital insights into the various interactions of omeprazole and the distinct nature of its connection with proteinoids.

**Table 2:** Examples of Omeprazole Inhibition Properties in Different Systems

system	inhibition properties	reference
proteinoids	suppression of potential spikes, reduction in amplitude, increase in period of spikes, concentration-dependent effects	this work
H^+^/K^+^-ATPase (proton pump)	inhibition of gastric acid secretion, increase in pH, reduction of proton transfer	(14,36)
cytochrome P450 enzymes	inhibition of drug metabolism, potential drug–drug interactions, altered pharmacokinetics	(37,38)
bacterial proton-gated ion channels	inhibition of proton-gated ion channels, reduction of bacterial virulence, potential antibacterial effects	(39,40)
acid-sensing ion channels (ASICs)	modulation of acid-sensing ion channels, potential neuroprotective effects, regulation of neuronal excitability	(41,42)

#### Capacitance Measurements of Omeprazole Proteinoid Sample: Insights into Electrical Properties

Dielectric relaxation spectroscopy is an effective method for studying the frequency-dependent characteristics of materials, offering valuable information about their molecular dynamics and structural features.^43^ The Debye model is a fundamental and extensively used approach among numerous models used to describe dielectric relaxation.^44^ The Debye relaxation model suggests that the polarization of a substance reacts to an electric field by displaying a unique relaxation period, leading to a distinct frequency-dependent response of the complex permittivity.^45^ The Debye equation relates the complex permittivity (ε*) to the frequency (ω) and the relaxation time (τ) as follows:
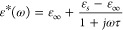
3where ε_*∞*_ is the high-frequency permittivity, ε_*s*_ is the low-frequency (static) permittivity, and *j* is the imaginary unit.^46^

The equation discusses the dispersion and absorption of electromagnetic waves within a substance, contingent upon frequency. It offers significant insights into the dielectric characteristics and molecular dynamics of the system.^47^ The Debye model has demonstrated its efficacy in several materials, including polar liquids, polymers, and biological systems.^48−50^ Nevertheless, many materials display deviations from the ideal Debye behavior, necessitating the use of complex models such as the Cole–Cole, Davidson–Cole, or Havriliak–Negami equations. These equations introduce supplementary parameters to take into account the broadening and asymmetry of the relaxation processes.^51,52^

**Figure 9:**
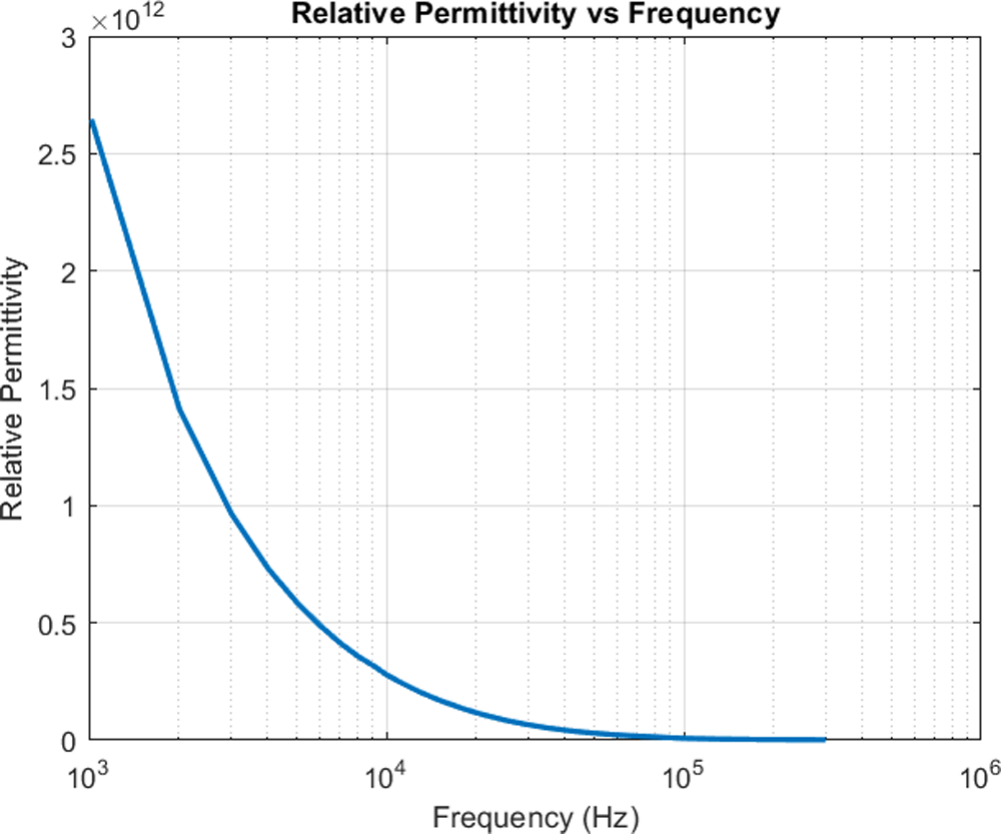
Frequency-dependent behavior of the relative permittivity for the omeprazole-proteinoid system is shown in this figure. The relative permittivity exhibits a decreasing trend with increasing frequency, which is characteristic of dielectric relaxation phenomena.

Figure 9 presents the frequency-dependent behavior of the relative permittivity (dielectric constant) for the omeprazole-proteinoid system. The relative permittivity was calculated from the measured capacitance using the equation:
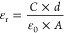
4where ε_*r*_ is the relative permittivity, *C* is the measured capacitance (F), *d* is the distance between the electrodes (m), ε_0_ is the permittivity of free space (F/m), and *A* is the area of the electrodes (m^2^). The calculation was performed using the following constants: ε_0_ = 8.85418782 × 10^–12^ F/m, *d* = 0.01 m, and the thickness of the needle-like electrodes is 0.002 m. The area of the electrodes was calculated using the formula for the area of a circle: *A* = π × (thickness/2)^2^. The relative permittivity is a dimensionless parameter that quantifies the ratio between the permittivity of a material and the permittivity of free space. The permittivity of a material and the permittivity of free space have the same units, which are Farads per meter (F/m). When their ratio is calculated, the units cancel out, resulting in a dimensionless quantity. The dimensionless property of relative permittivity enables the comparison of dielectric properties among different materials, independent of units.

The statistical analysis of the relative permittivity values shows a broad range, ranging from a minimum value approaching 0 at high frequencies to a maximum value of approximately 2.5 × 10^12^ at low frequencies. The relative permittivity exhibits a strong frequency dependence, showing a characteristic decrease as frequency increases from 10^3^ to 10^6^ Hz. The most significant changes occur in the lower frequency range (10^3^–10^4^ Hz), where the relative permittivity decreases rapidly from 2.5 × 10^12^ to approximately 5 × 10^11^. The relative permittivity values reported for the omeprazole-proteinoid system exhibit a significant increase when compared to the values for several other materials. As an example, water has a relative permittivity of around 80 at ambient temperature,^53^ but common polymers like polyethylene and polypropylene have a relative permittivity ranging from 2 to 3.^54^ The omeprazole-proteinoid system has a high relative permittivity, indicating a robust polarization response to the electric field. This can be due to the distinctive molecular structure and interactions present in the system. Various systems in the field of biomaterials have been examined for their dielectric characteristics. In their study, Bang et al.^55^ examined the dielectric characteristics of silk fibroin films and observed relative permittivity values between 5 and 7 at room temperature, which varied according to the frequency. In an additional study conducted by Kovacic et al.^56^ the dielectric characteristics of a chitosan-starch composite film were analyzed. The study revealed that the relative permittivity values varied between 10 and 100 when measured at low frequencies ranging from 1 to 100 Hz. Although the results are lower compared to the omeprazole-proteinoid system, they illustrate the wide range of dielectric characteristics displayed by biomaterials. The increased relative permittivity of the omeprazole-proteinoid mixture has significant implications for its prospective uses. Materials having higher dielectric constants are preferred for a range of applications, including energy storage devices, capacitors, and electronic packaging.^57^ Furthermore, the Figure 9 illustrates the way in which the relative permittivity changes with frequency, offering valuable information about the dielectric relaxation mechanisms present in the omeprazole-proteinoid system. The decrease in relative permittivity as frequency increases is in line with the expected behavior of dielectric materials, where the polarization response lags behind the applied electric field at higher frequencies.^43^

**Figure 10:**
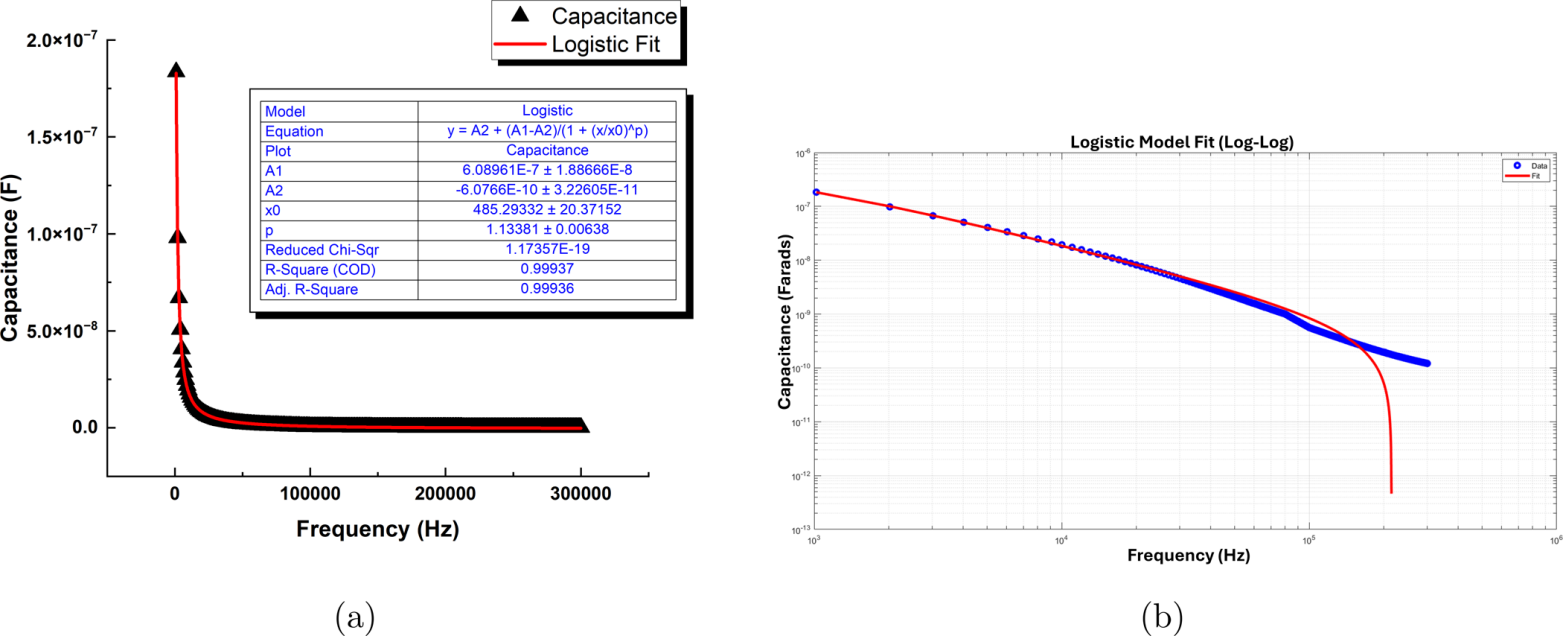
(a) The capacitance (in Farads) of the omeprazole-proteinoid system is plotted against the frequency (in Hz). The experimental data is fitted using the Logistic model, represented by the equation: . The best-fit parameters are *A*_1_ = (6.08961 ± 1.88666) × 10^–7^, *A*_2_ = (−6.0766 ± 3.22605) × 10^–10^, *x*_0_ = 485.29332 ± 20.37152, and *p* = 1.13381 ± 0.00638. The Logistic model shows an excellent agreement with the experimental data, as evidenced by the high *R*-square (COD) value of 0.99937 and the low reduced chi-square value of 1.17357 × 10^–19^. The adjusted *R*-square value of 0.99936 further confirms the goodness of fit. (b) The log–log plot of capacitance versus frequency reveals the frequency-dependent behavior of the omeprazole-proteinoid system. The Debye relaxation analysis is performed based on the Logistic model parameters. The low-frequency (static) permittivity (ε_s_) is found to be 6.0896 × 10^–7^ F/m, while the high-frequency permittivity (ε_*∞*_) is −6.0766 × 10^–10^ F/m. The negative value of the high-frequency permittivity is likely due to the limitations of the Logistic model in capturing the high-frequency behavior accurately. The relaxation time (τ), which characterizes the time scale of the polarization response, is determined to be 3.2796 × 10^–4^ s. The Logistic model parameters (*A*_1_, *A*_2_, *x*_0_, and *p*) obtained from the fitting are also reported.

Figure 10 presents the capacitance versus frequency analysis of the omeprazole-proteinoid system using the Logistic model and Debye relaxation theory. The experimental data, shown in Figure 10a, is fitted using the Logistic model, which is represented by the eq 5:
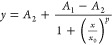
5The best-fit parameters obtained from the Logistic model are *A*_1_ = (6.08961 ± 1.88666) × 10^–7^, *A*_2_ = (−6.0766 ± 3.22605) × 10^–10^, *x*_0_ = 485.29332 ± 20.37152, and *p* = 1.13381 ± 0.00638. The excellent agreement between the Logistic model and the experimental data is evidenced by the high *R*-square (COD) value of 0.99937 and the low reduced chi-square value of 1.17357 × 10^–19^. The adjusted *R*-square value of 0.99936 further confirms the goodness of fit, indicating that the Logistic model accurately describes the capacitance behavior of the omeprazole-proteinoid system over the investigated frequency range.^50,58^ The log–log plot of capacitance versus frequency, presented in Figure 10b, reveals the frequency-dependent behavior of the system. In order to calculate the Debye relaxation parameters from the fitted parameters of the Logistic model, the following equations were employed. The low-frequency (static) permittivity (ε_s_) was obtained directly from the fitted parameter *A*_1_ of the Logistic model, as given by
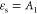
6Similarly, the high-frequency permittivity (ε_*∞*_) was assigned the value of the fitted parameter *A*_2_ from the Logistic model:
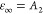
7The relaxation time (τ), which characterizes the time scale of the polarization response, was calculated using the equation:
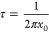
8where *x*_0_ is the fitted parameter from the Logistic model. These calculations allow for the determination of the Debye relaxation parameters based on the fitted parameters of the Logistic model, providing insights into the dielectric properties of the omeprazole-proteinoid system. The low-frequency permittivity represents the static dielectric constant of the system, the high-frequency permittivity corresponds to the dielectric constant at infinite frequency, and the relaxation time characterizes the time scale of the polarization response.

The Debye relaxation analysis, based on the Logistic model parameters, yields a low-frequency (static) permittivity (ε_s_) of 6.0896 × 10^–7^ F/m and a high-frequency permittivity (ε_*∞*_) of −6.0766 × 10^–10^ F/m. The negative value of the high-frequency permittivity suggests limitations of the Logistic model in capturing the high-frequency behavior accurately.^51^ The relaxation time (τ), which characterizes the time scale of the polarization response, is determined to be 3.2796 × 10^–4^ s.

The Debye relaxation analysis provides valuable insights into the dielectric properties of the omeprazole-proteinoid system.^46^ The low-frequency permittivity represents the static dielectric constant of the system, while the high-frequency permittivity corresponds to the dielectric constant at infinite frequency.^44^ The relaxation time indicates the characteristic time scale over which the polarization of the system responds to the applied electric field.^59^ The log–log representation of the capacitance versus frequency data allows for a clear visualization of the frequency-dependent behavior over a wide range of frequencies.^49^ The Logistic model captures the overall trend of the capacitance data, including the low-frequency plateau and the high-frequency dispersion. However, the negative value of the high-frequency permittivity suggests that the Logistic model may have limitations in accurately describing the high-frequency behavior of the system.^52^

Additional insights into the dielectric properties of the omeprazole-proteinoid system may be gained through further experiments, such as the exploration of different dielectric relaxation models or the consideration of the physical mechanisms that underlie the relaxation process.^43,48^ The Debye relaxation analysis, using the Logistic model, provides a basis for understanding the frequency-dependent behavior and time scales that define the polarization response of the system.

#### Impedance Spectroscopy Analysis and Equivalent Circuit Modeling

**Figure 11:**
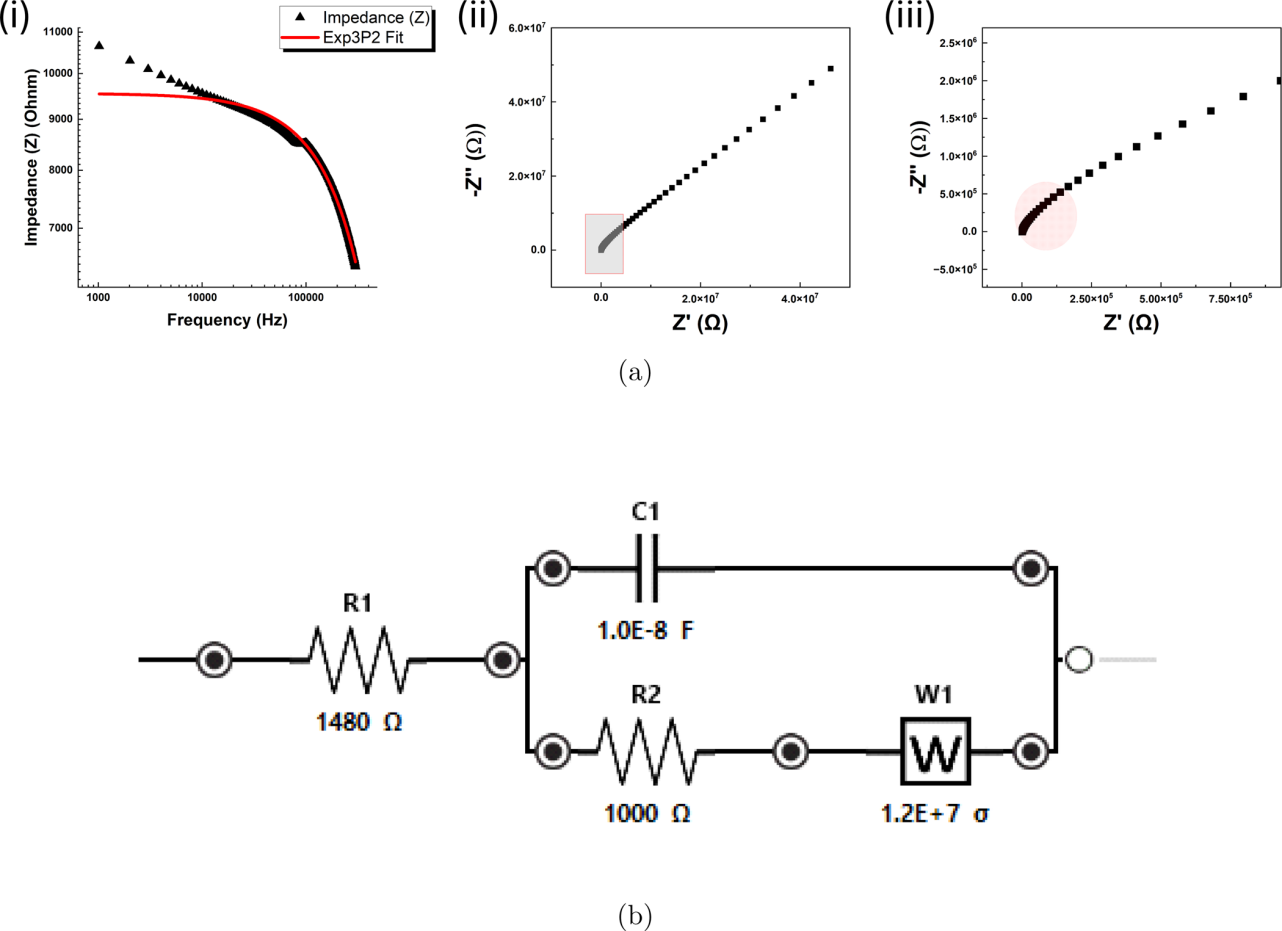
Impedance spectroscopy analysis of the omeprazole-proteinoid system using the Exp3P2 model, and the Randles circuit model. (a) (i) Exp3P2 model fit of the impedance data on a log–log scale. (ii) Nyquist plot of the experimental data and the fitted models. The impedance data was analyzed using the Exp3P2 model, and the Randles circuit model. The Exp3P2 model, described by the equation *Z* = exp(*a* + *b* × *f* + *c* × *f*^2^), where *Z* is the impedance and *f* is the frequency, was fitted to the experimental data. The fitted parameters for the Exp3P2 model were found to be *a* = 9.16432, *b* = −1.13394 × 10^–6^, and *c* = −5.30867 × 10^–13^. The Nyquist plot displays the real part of the impedance against the negative imaginary part. The experimental data points follow a semicircular shape, indicating the presence of resistive and capacitive elements in the system. The Randles circuit model, which consists of a solution resistance (*R*_s_) in series with a parallel combination of a double-layer capacitance (*C*_dl_) and a faradaic impedance (*Z*_f_), was fitted to the impedance data. The fitted parameters for the Randles circuit model were *R*_s_ = 1.48 × 10^3^ Ω, *C*_dl_ = 6.95 × 10^3^ F, *R*_ct_ = 9.95 × 10^4^ Ω, and *Z*_w_ = 1.23 × 10^4^ Ω *s*^–1/2^, where *R*_ct_ is the charge transfer resistance and *Z*_w_ is the Warburg impedance. The Randles circuit model fit aligns well with the experimental data in the Nyquist plot, capturing the semicircular shape and the diffusion-related linear region at lower frequencies. (ii) (iii) Enlargement of the Nyquist plot (b) Randles circuit containing capacitance, resistances, and a Warburg element (W).

Impedance spectroscopy was employed to investigate the electrical properties of the omeprazole-proteinoid system. The impedance data was analyzed using two different models: the Exp3P2 model and the Randles circuit model. The Exp3P2 model, described by the equation
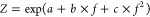
9where *Z* is the impedance and *f* is the frequency, was fitted to the experimental data. The fitted parameters for the Exp3P2 model were found to be *a* = 9.16432, *b* = −1.13394 × 10^–6^, and *c* = −5.30867 × 10^–13^. The impedance values calculated using the Exp3P2 model are shown in Figure 11a(i) on a log–log scale. The Nyquist plot, presented in Figure 11a(ii), displays the real part of the impedance against the negative imaginary part. The experimental data points follow a semicircular shape, indicating the presence of resistive and capacitive elements in the system. An enlargement of the Nyquist plot is provided in Figure 11a(iii). The Randles circuit model, which consists of a solution resistance (*R*_s_) in series with a parallel combination of a double-layer capacitance (*C*_dl_) and a faradaic impedance (*Z*_f_), was also employed to analyze the impedance data. The fitted parameters for the Randles circuit model were *R*_s_ = 1.48 × 10^3^ Ω, *C*_dl_ = 6.95 × 10^3^ F, *R*_ct_ = 9.95 × 10^4^ Ω, and *Z*_w_ = 1.23 × 10^4^ Ω s^–1/2^, where *R*_ct_ is the charge transfer resistance and *Z*_w_ is the Warburg impedance. The Randles circuit model fit aligns well with the experimental data in the Nyquist plot, capturing the semicircular shape and the diffusion-related linear region at lower frequencies. The schematic of the Randles circuit, containing capacitance, resistances, and a Warburg element (W), is shown in Figure 11b.

#### Boolean Logic Interactions in Proteinoid-Omeprazole Complexes

**Figure 12:**
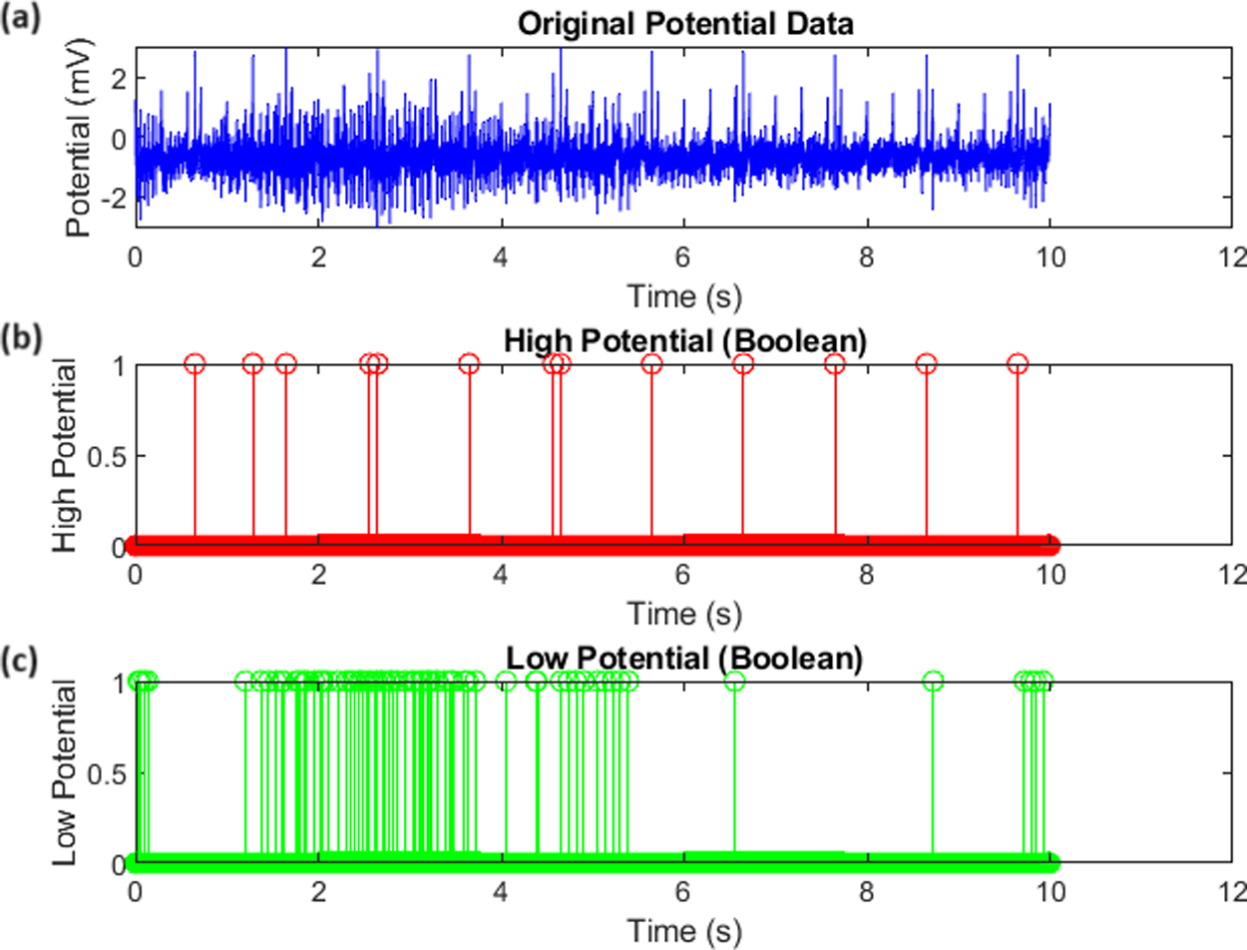
Boolean representation of the proteinoid-omeprazole interaction. (a) Original potential data. (b) High potential state (red stem plots). (c) Low potential state (green stem plots).

The interaction between proteinoids and omeprazole can be modeled using Boolean logic, which allows for a simplified representation of the complex biochemical processes involved. In this approach, we define two threshold values, threshold_high_ and threshold_low_, to categorize the potential data into high and low states. The high potential state is represented by the Boolean variable high_potential_, which is true when the potential exceeds threshold_high_, as shown in eq 10. Similarly, the low potential state is represented by the Boolean variable low_potential_, which is true when the potential falls below threshold_low_, as shown in eq 11.
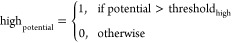
10
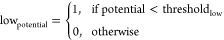
11The Boolean representation of the proteinoid-omeprazole interaction allows for a clear visualization of the system’s behavior. Figure 12 displays the original potential data, along with the corresponding high and low potential states. The high potential state is represented by red stem plots, indicating the time points at which the potential exceeds threshold_high_. In contrast, the low potential state is represented by green stem plots, indicating the time points at which the potential falls below threshold_low_. This visualization provides a concise overview of the system response to the interaction between proteinoids and omeprazole. The Boolean approach to modeling the proteinoid-omeprazole interaction has several advantages. First, it simplifies complex biochemical processes into a binary representation, making it easier to analyze and interpret the behavior of the system. Second, it allows for the identification of critical time points at which the system exhibits significant changes in potential, which can be further investigated to gain insights into the underlying mechanisms. Finally, the Boolean representation can be easily integrated into computational models and simulations, enabling the exploration of various scenarios and hypotheses related to the proteinoid-omeprazole interaction.

The Table 3 presents the application of various Boolean logic gates to the potential data, providing valuable insights into the temporal patterns and relationships between the high and low potential states.

**Table 3:** Application of Boolean Logic Gates to Potential Data^a^

time (s)	high	low	AND	OR	XOR	NOT	NAND	NOR	XNOR
0.000100	0	0	0	0	0	1	1	1	1
0.000200	0	0	0	0	0	1	1	1	1
0.000300	0	0	0	0	0	1	1	1	1
⋮	⋮	⋮	⋮	⋮	⋮	⋮	⋮	⋮	⋮
0.222200	0	0	0	0	0	1	1	1	1
0.222300	0	0	0	0	0	1	1	1	1

a The table presents the outputs of various Boolean logic operations performed on the high and low potential states derived from the original potential data. The high potential state (High) is determined by comparing the potential values against a threshold of 2 mV, while the low potential state (Low) is determined by comparing against a threshold of −2 mV. The Boolean operations performed include AND, OR, XOR, NOT, NAND, NOR, and XNOR. The AND operation returns 1 only when both High and Low states are 1, indicating the simultaneous occurrence of high and low potential states. The OR operation returns 1 when either High or Low state is 1, capturing the presence of either high or low potential. The XOR operation returns 1 when either High or Low state is 1, but not both, highlighting the exclusive occurrence of high or low potential. The NOT operation inverts the High state, returning 1 when the potential is below the high threshold. The NAND operation is the inverse of the AND operation, returning 0 only when both High and Low states are 1. The NOR operation is the inverse of the OR operation, returning 1 only when both High and Low states are 0. The XNOR operation is the inverse of the XOR operation, returning 1 when both High and Low states are either 0 or 1.

**Figure 13:**
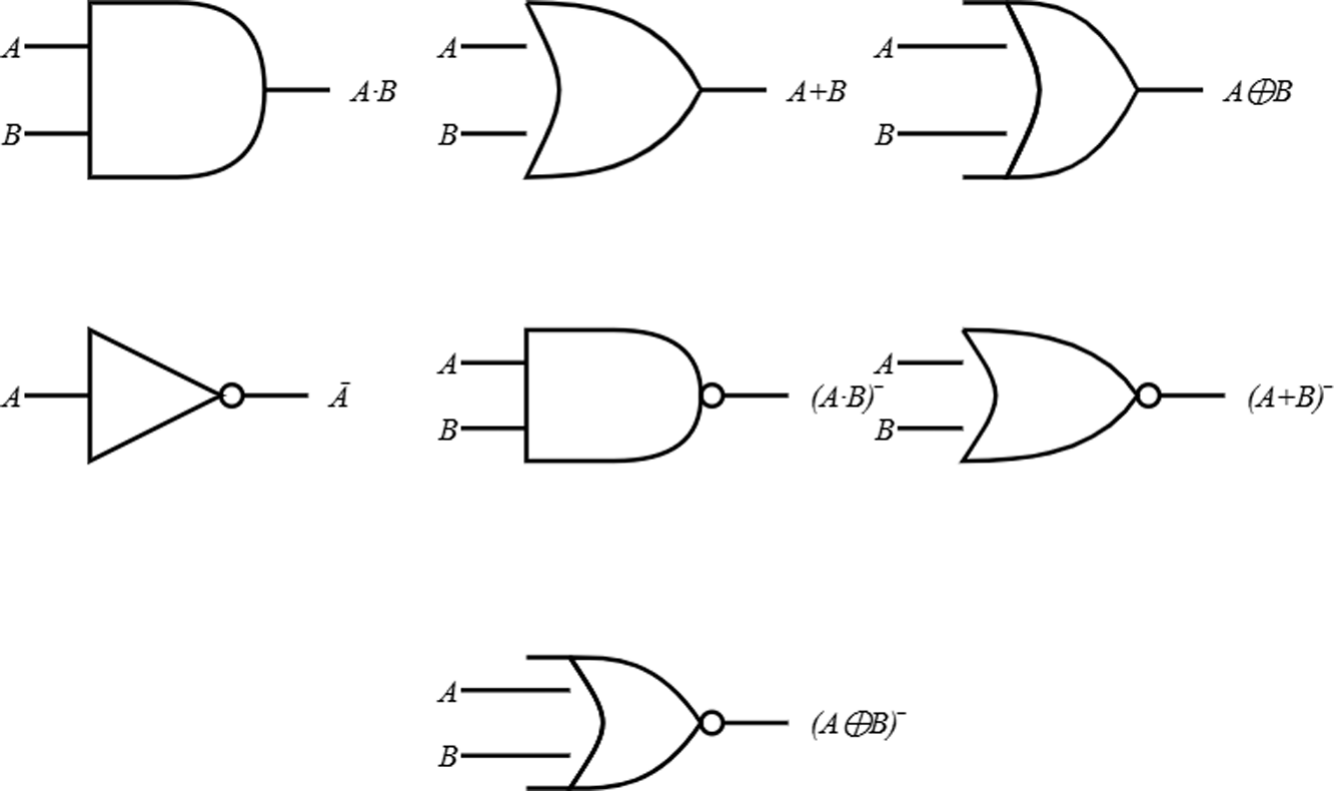
Logic gate diagrams: AND, OR, XOR, NOT, NAND, NOR, and XNOR.

These Boolean operations, including AND, OR, XOR, NOT, NAND, NOR, and XNOR, can be mathematically represented using equations, as shown in Figure 13. The AND operation, denoted as *A* × *B*, returns 1 only when both inputs (A and B) are 1. In the context of the potential data, the AND operation captures the simultaneous occurrence of high and low potential states. Mathematically, it can be expressed as
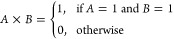
12The OR operation, represented as *A* + *B*, returns 1 when either input (A or B) is 1. In the table, the OR operation indicates the presence of either high or low potential states. The equation for the OR operation is
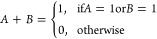
13The XOR (exclusive OR) operation, denoted as *A* ⊕ *B*, returns 1 when either input is 1, but not both. In the potential data analysis, the XOR operation highlights the exclusive occurrence of high or low potential states. The XOR equation is
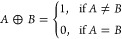
14The NOT operation, represented as *A̅*, inverts the input. In the table, the NOT operation is applied to the high potential state, returning 1 when the potential is below the high threshold. The NOT equation is
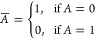
15The NAND (NOT AND) operation, denoted as , is the inverse of the AND operation. It returns 0 only when both inputs are 1. The NAND equation is
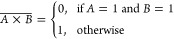
16The NOR (NOT OR) operation, represented as , is the inverse of the OR operation. It returns 1 only when both inputs are 0. The NOR equation is
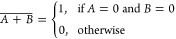
17The XNOR (exclusive NOR) operation, denoted as , is the inverse of the XOR operation. It returns 1 when both inputs are either 0 or 1. The XNOR equation is
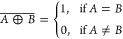
18It is important to note that these Boolean logic operations have substantial consequences in unconventional computing, particularly in the field of reservoir computing and neuromorphic systems. Through the application of these processes to the prospective data, as shown in the table, it is possible to extract and analyze complex temporal patterns and correlations. This enables the creation of new computational models that utilize the dynamic and nonlinear characteristics of physical systems, such the one represented by the potential data. When it comes to the construction of more complex computational models and architectures, the Boolean logic gates make up the essential building blocks. For the purpose of simulating the operation of biological neural networks, it is possible to accomplish complex information processing tasks by combining these gates in a variety of combinations. The Table 3 demonstrates the potential of employing Boolean logic operations to find underlying patterns and relationships within the prospective data. This opens the door for the construction of unconventional computing systems that are both efficient and powerful. Figure 13 provides a visual representation of the logic gates, which, when combined with the mathematical equations that represent these processes, provide a thorough knowledge of the functionality and implications of these gates.

#### Proton-Motive Force and the Potential Role of Proteinoids in the Origin of Life

The proton-motive force, a key principle in the field of biology, serves as a vital component in energising life on our planet.^60^ The constant electric field, created by the movement of protons across membranes, is the primary catalyst for the metabolic activities that support every living organism. It proposes that natural proton gradients in hydrothermal vents^61,62^ may have facilitated the development of early biochemical reactions and cell-like structures. The idea becomes especially captivating when considering the possible contribution of proteinoids, like the ones examined in our study of the proteinoid-omeprazole system, as prebiotic compounds that may have played a part in the emergence of life.^63^ The hydrothermal vent hypothesis suggests that the combination of hydrogen and carbon dioxide,^64^ fueled by natural proton gradients across thin inorganic barriers, may have resulted in the creation of organic molecules within cell-like pores. This hypothesis bears similarities to the interactions we saw between proteinoids and omeprazole in our research. In this context, the proton-motive force and electrical charges across membranes play a crucial role in inhibiting potential spikes and modulating the behavior of the system. Although the hydrothermal vent concept and our proteinoid-omeprazole system may have different mechanisms and timeframes, they both rely on the fundamental principles of proton gradients and electrical charges to drive metabolic processes. Proteinoids, which are primordial molecules capable of forming cell-like structures and displaying complex behaviors, have been extensively regarded as probable constituents of early life. This emphasizes the necessity for alternative methods that take into account the involvement of cell-like structures and the proton-motive force.^65^ The analysis of the proteinoid-omeprazole system aims to understand how these prebiotic molecules interact with a proton pump inhibitor. This research has the potential to offer significant knowledge about the basic principles that regulate the development and origins of life.^66−68^ Living organisms use proton pumps to actively transfer protons across membranes, and this process requires energy, such as ATP. This leads to the inquiry into the mechanism by which proton pumps function in proteinoid membranes, considering that proteinoids lack the natural energy sources present in living systems. If we assume the existence of proton pumps in proteinoid membranes, the specific mechanism by which they function is yet unknown and requires additional research. There is a possibility that these primitive systems could be driven by alternative energy sources or chemical gradients through proton pumping, although this is currently based on speculation. Additional research is required to examine the efficiency and underlying mechanisms of proton pumps in relation to proteinoid membranes and their role in to the emergence of life.

Hydrothermal vents on the seafloor may be where life began. They provide energy, nutrients, and a safe place for chemical processes. These may have led to the first self-sustaining metabolic cycles. These systems have steep temperature and chemical gradients. They support diverse microbes that drive key biogeochemical cycles.^69^ Recent studies have revealed the microbial sulfur cycle. They also showed the role of microbes in transforming methane in hydrothermal vent ecosystems. Microbial oxidation of methane, a potent greenhouse gas, helps. It keeps methane out of the atmosphere and traps carbon in seafloor minerals. The microbial sulfur cycle in low-temperature hydrothermal vents can also create valuable minerals.^70^ The Guaymas Basin in the Gulf of California is a hydrothermal system. It supports diverse microbes that can process various carbon and energy sources. Here, steep temperature (from near-freezing seawater to fluids exceeding 400 °C) and geochemical gradients create niches for microbes. They are involved in methane cycling and sulfate reduction. This may couple the oxidation of abundant alkanes to sulfate reduction. Hydrothermal vents are vital to global biogeochemical cycles. They also offer rich resources, from fisheries to bioprospecting.^71^ Studying the microbes that sustain these unique ecosystems can offer insights into the origins of life on Earth. It may also help us understand the potential for life elsewhere in the universe.^72^

Two processes connect hydrothermal vents to the emergence of life. First, hydrothermal vents create proton gradients.^73^ This generates electrochemical potential differences, like those in modern cellular energy systems. These gradients could have provided the initial energy source for prebiotic chemical reactions. Second, proteinoids are thermal proteins. They form from amino acids under prebiotic conditions. They show properties that bridge the gap between nonliving and living systems. They can form microspheres reminiscent of primitive cells and exhibit basic catalytic activities. These processes are linked by proton-motive force. It is vital to all known life. Modern cellular systems, including our experimental proteinoid-omeprazole system, use proton gradients for energy. This suggests an ancient origin for the mechanism. This framework links early Earth’s hydrothermal vents with the chemical evolution of proteinoids. They both depend on proton-driven energy.

## Experimental Section

### Synthesis of l-Glutamic Acid:l-Aspartic Acid:l-Phenylalanine (l-Glu:l-Asp:l-Phe) Proteinoids

The proteinoid synthesis method used high-purity amino acids obtained from Sigma-Aldrich, specifically l-Glutamic acid (≥99%, CAS Number: 56-86-0), l-Aspartic acid (≥98%, CAS Number: 56-84-8), and l-Phenylalanine (≥98%, CAS Number: 63-91-2). The synthesis approach used deionized water having a resistivity of at least 18.2 MΩ cm, which was obtained using a Millipore water purification system. All compounds were used in their original form without undergoing additional purification. The proteinoid microspheres were synthesized using a multistep thermal polymerization and precipitation process. The amino acids l-Glutamic acid, l-Aspartic acid, and l-Phenylalanine were mixed together in a 1:1:1 molar ratio (2.5 g each) inside a 50 mL round-bottom flask. Next, the flask was attached to a reflux condenser and heated to a temperature of 300 °C using a Stuart heat-stir hot plate magnetic stirrer equipped with temperature control. The amino acid mixture underwent continuous agitation at a speed of 500 rpm for a duration of 3 h, facilitating thermal poly condensation and resulting in the formation of a thermally polymerized product. Upon reaching room temperature, the proteinoid mixture was completely dissolved in deionized water to obtain a concentration of 1 mg/L. The proteinoid solution was subjected to a temperature of 80 °C and kept at this level for an extra 3 h while being continuously stirred at a speed of 500 rpm. The secondary heating step promoted the precipitation and production of the synthesized proteinoids microspheres. After the second heating procedure, the solution was cooled to to room temperature and subjected to lyophilization using a BIOBASE model BK-FD10P Freeze-Dry System in order to obtain the solid proteinoid. The proteinoid was ground into a fine powder using a mortar and pestle and then kept in a desiccator for later use.

### Preparation of Omeprazole-Proteinoid Mixture

The omeprazole-proteinoid mixture was synthesized via preparing individual solutions of omeprazole and proteinoid, and then mixing the solutions to make the final formulation. Three omeprazole samples were produced by dissolving various amounts of omeprazole (7.2, 8.3, and 13 mg) in 5 mL of dimethyl sulfoxide (DMSO) from Sigma-Aldrich (CAS Number: 67-68-5, EC Number: 200-664-3, Molecular Weight: 78.13 g/mol). An analytical balance was used to weigh the omeprazole (Merck, CAS Number: 73590-58-6, Molecular Weight: 345.42 g/mol), which was then transferred to clean and dry beakers. The mixtures were subsequently stirred using a magnetic stirrer at ambient temperature until the omeprazole was fully dissolved. A 5 mL proteinoid solution containing l-Glutamic Acid (l-Glu), l-Phenylalanine (l-Phe), and l-Aspartic Acid (l-Asp) has been made in a separate beaker. The proteinoid was well dissolved in the water solution. The omeprazole solutions, which were made in DMSO, were introduced gradually to the beaker that contained the proteinoid solution in water. The solutions were thoroughly mixed by gently stirring them with a magnetic stirrer. The stirring process was prolonged for a duration of 5–10 min in order to ensure thorough and complete mixing of the omeprazole-proteinoid blends. The synthesized omeprazole-proteinoid mixtures were then ready for further characterization and measurements.

### Electrochemical Characterization of the Proteinoid-Omeprazole System

The experimental configuration comprises of two needle electrodes, fabricated from platinum and iridium coated stainless steel wires (Spes Medica S.r.l., Italy), that are submerged in the proteinoid-omeprazole sample solution and positioned at a distance of 10 mm from each other. The electrodes are linked to a high-precision 24-bit ADC data recorder (Pico Technology) for the purpose of capturing voltage responses with great sensitivity. This allows for the identification of even the smallest voltage variations in the microvolt range. The provided solution is placed within a container positioned on a heating block, which enables accurate regulation and observation of the temperature throughout the experiment. The heating block and the data logger are synchronized to enable the simultaneous capture of thermal and electrical characteristics. Through electrodes, a BK Precision 4053 MHz dual channel waveform generator supplies electrical stimuli imitating thalamocortical Izhikevich neurons to the proteinoid-omeprazole solution. Finally, a Rigol oscilloscope (2 Channel 100 MHz–1GSa/s), PicoLog ADC-24, and Picoscope capture data, together with a Keithley 2450 sourcemeter for electrical measurements. This experimental configuration allows for the visualization of voltage responses in the proteinoid-omeprazole system, providing essential data about the electrochemical behavior and potential changes of this unique combination under different experimental settings.

### Morphological Characterization of Proteinoid-Omeprazole Complexes

We investigated the morphological properties of the proteinoid-omeprazole complexes using an FEI Quanta 650 Field Emission Scanning Electron Microscope (FE-SEM). This advanced imaging technique allows for the capture of high-resolution images of the sample surface, offering valuable insights into the structural features and composition of the proteinoid-omeprazole complexes at the micro and nanoscale. In order to analyze the samples using FE-SEM, a thin layer of gold was applied to the proteinoid-omeprazole complexes using a sputter coater. The gold coating has two key functions. First, it acts as a protective barrier, preserving the sample from damage during imaging. Second, it improves the conductivity of the sample surface, allowing for the formation of a stable charged-particle beam necessary for high-resolution imaging. The FEI Quanta 650 FE-SEM was operated in a high vacuum environment, using an accelerating voltage of 5–10 kV and a working distance of 8–10 mm. The microscope’s electron optics and detectors enabled the capture of highly detailed surface morphology images at a wide range of magnifications, from 1000× to 100,000×.

## Conclusions

Our study investigates the effect of the proton pump inhibitor omeprazole on the spiking features of proteinoids, with potential implications for unconventional computing. Through our experimentation, we have observed that omeprazole appears to modify the size, rate, and timing of proteinoid spikes, though these changes are subtle (0.1–0.2 mV) but detectable within our measurement capabilities (PicoLog ADC-24 resolution of 12 μV). This finding provides us with a valuable means of manipulating and directing the behavior of these interesting biomolecules. Our findings indicate that the flow of protons across the proteinoid membrane may be the main cause of electrical spiking in these primitive protein structures.This result reveals the origins and nature of electrical signaling in proteinoids and the potential role of proton gradients in bioelectrical phenomena in early life-like systems. By integrating Boolean logic operations with the proteinoid-omeprazole system, we have successfully revealed complex interactions and hidden patterns within the spiking data. This has led to a deeper understanding of the fundamental mechanisms that control the behavior of proteinoids. The discovery has significant implications for the creation of new approaches to computing and architectures that use the inherent nonlinearity and complexity of biological systems. Our findings challenge the conventional boundaries of computing and showcase the immense potential of proteinoids as a platform for unconventional computing. Using the shifts in proteinoid spiking caused by omeprazole, we may imagine a future in which biologically inspired computing systems, which can adapt, learn, and solve complex problems, become a real possibility. Nevertheless, our efforts are merely the starting point. Additional research is required to thoroughly examine the potential of proteinoids and the impact of different biochemical agents on their spiking behavior.

## Supporting Information Available

Figure S1: analysis of potential spikes in omeprazole-proteinoid mixtures at two concentrations. At 0.72 mg/mL, spikes show higher amplitudes (mean 1.91 mV) with lower variability in period (mean 4285.28 s). At 0.83 mg/mL, spikes have lower amplitudes (mean 0.46 mV) but show greater period variability (mean 6461.62 s). Spikes were detected using MATLAB with a 0.1 mV threshold and 10000-s minimum peak distance; Figure S2: visualization of potential dynamics and spike periodicity for two concentrations, presents time-series potential data with marked spikes (red triangles) and corresponding period distributions for both 0.72 and 0.83 mg/mL concentrations, and shows how concentration affects both spike characteristics and temporal patterns in the omeprazole-proteinoid system (PDF)
