# Supplementary material for: Proton Pump Inhibitor Omeprazole Alters the Spiking Characteristics of Proteinoids
Source: ACS Omega. 2025 Jan 27;10(5):5016–35. doi: 10.1021/acsomega.4c10790 (PMC11822715; doi:10.1021/acsomega.4c10790)
Supplement: Supplementary file 1 — ao4c10790_si_001.pdf [file ao4c10790_si_001.pdf]

Supporting Information

Proton Pump Inhibitor Omeprazole Alters the Spiking Characteristics of Proteinoids

Panagiotis Mougkogiannis[1,*] and Andrew Adamatzky[1]

[1]Unconventional Computing Laboratory, University of the West of England, Bristol, UK

Email: Panagiotis.Mougkogiannis@uwe.ac.uk

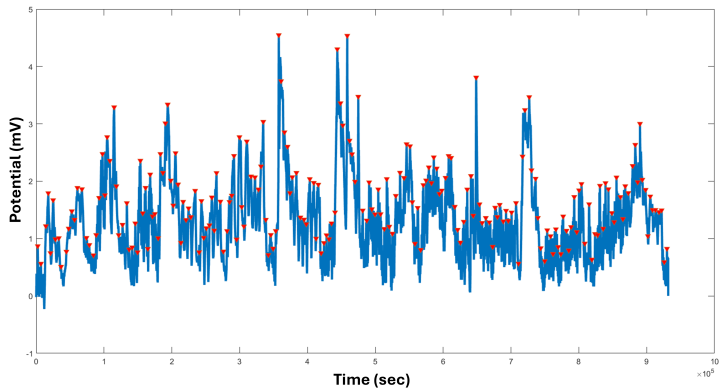

(a)

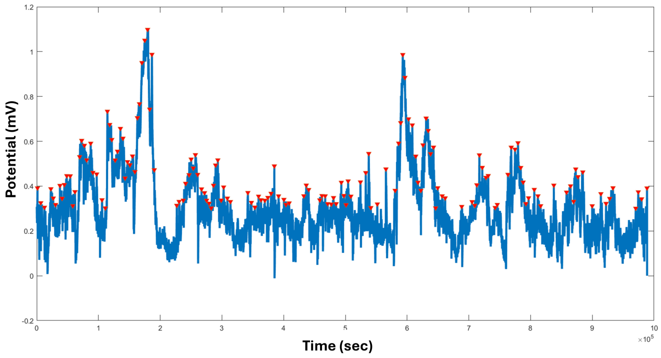

(b)

Figure S1: Potential (mV) versus time (sec) for omeprazole-proteinoid at concentrations of (a) 0.72 mg/ml and (b) 0.83 mg/ml. The potential spikes, automatically detected using a MATLAB program with a threshold of 0.1 mV and a minimum peak distance of 10000 seconds, are marked with red inverted triangles. For the 0.72 mg/ml concentration, the amplitude statistics are as follows: quartiles (1.37 mV, 1.77 mV, 2.27 mV), mean (1.91 mV), max (4.78 mV), min (0.74 mV), and standard deviation (0.75). The period statistics for the 0.72 mg/ml concentration are: quartiles (3443.50 sec, 3948.00 sec, 4884.50 sec), mean (4285.28 sec), max (7846.00 sec), min (2196.00 sec), and standard deviation (1002.60 sec). For the 0.83 mg/ml concentration, the amplitude statistics are: quartiles (0.35 mV, 0.41 mV, 0.53 mV), mean (0.46 mV), max (1.11 mV), min (0.31 mV), and standard deviation (0.16 mV). The period statistics for the 0.83 mg/ml concentration are: quartiles (3683.00 sec, 4606.00 sec, 6084.75 sec), mean (6461.62 sec), max (36858.00 sec), min (1209.00 sec), and standard deviation (5664.97 sec). The potential spikes and their statistical characteristics provide insights into the electrochemical behaviour and stability of the omeprazole-proteinoid system at different concentrations.

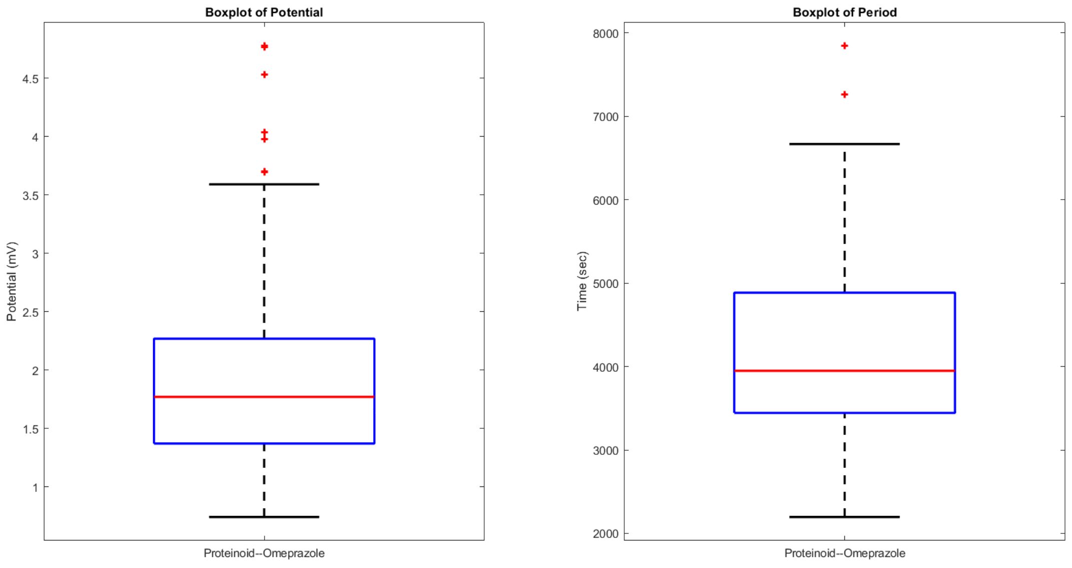

(a)

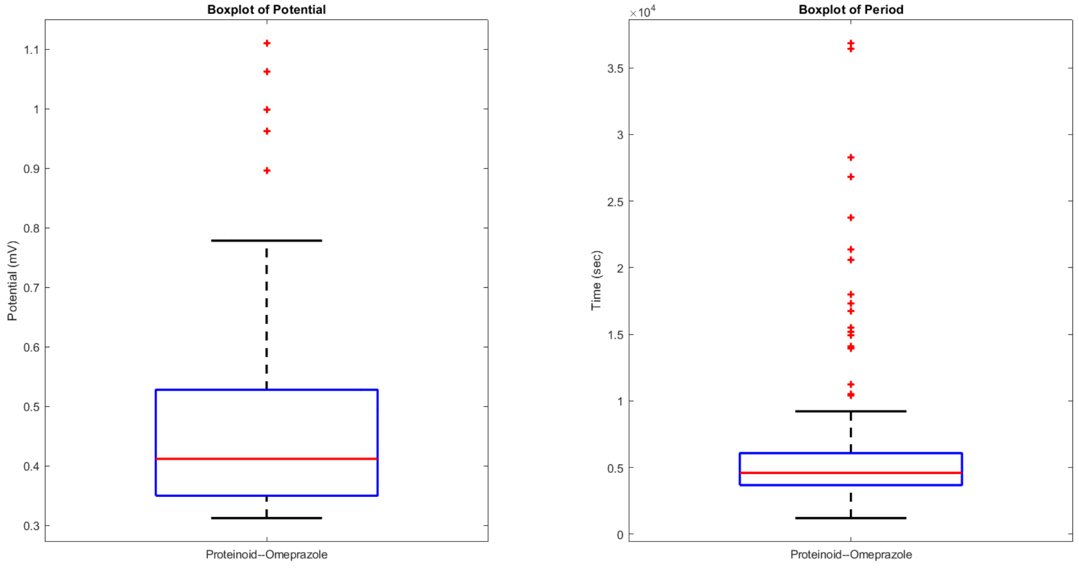

(b)

Figure S2: Potential (mV) versus time (sec) and periods (sec) for omeprazole-proteinoid at concentrations of 0.72 mg/ml and 0.83 mg/ml. (a) The potential plot for 0.72 mg/ml concentration shows the variation of potential over time, with potential spikes marked by red inverted triangles. The periods plot for 0.72 mg/ml concentration displays the distribution of time intervals between consecutive potential spikes. (b) The potential plot for 0.83 mg/ml concentration shows the potential behavior over time, with potential spikes indicated by red inverted triangles. The periods plot for 0.83 mg/ml concentration represents the distribution of time intervals between successive potential spikes. These plots provide insights into the electrochemical dynamics and stability of the omeprazole-proteinoid system at different concentrations, highlighting the differences in potential behavior and spike periodicity.
